# Multidisciplinary approaches to lithological discrimination and structural mapping for mineral resource assessment

**DOI:** 10.1038/s41598-026-43824-x

**Published:** 2026-03-13

**Authors:** Mohamed Abdelkawy Elfadly, Mohamed Abdelrady, Alessandro Decarlis, Ali A. Khudeir, Mohamed Ali Abu El-Rus, Hassan Abbas, Hany H. El Hadek, Ismael M. Ibraheem, Ali Shebl

**Affiliations:** 1 R.I.C.H. Center: Research and Innovation on CO2 and H2, Khalifa University of Science and Technology, P.O. Box 127788, Abu Dhabi, UAE; 2 Earth Sciences Department, Khalifa University of Science and Technology, P.O. Box 127788, Abu Dhabi, UAE; 3 Geology Department, Faculty of Science, Assiut University, Assiut, 71516 Egypt; 4 Department of Mineralogy, Institute of Geography and Earth Sciences, Eötvös Loránd University, Budapest, 1117 Hungary; 5 Institute of Geophysics and Meteorology, University of Cologne, Pohligstrasse 3, 50969 Cologne, Germany; 6 Department of Geology, Tanta University, Tanta, 31527 Egypt; 7 Department of Mineralogy and Geology, University of Debrecen, Egyetem tér 1, Debrecen, 4032 Hungary

**Keywords:** Aeromagnetic, Remote sensing, Wadi shait, Center for exploration targeting, Shait granite complex, 3D magnetic modeling, Edge detection, Environmental sciences, Solid Earth sciences

## Abstract

Understanding the geological and structural controls on mineralization is globally important for sustainable resource exploration and management. This study investigates the Gardan Ophiolitic Mélange (GOM) and the Shait Granite Complex (SGC) in the Wadi Shait area, Eastern Desert, Egypt, using an integrated, multidisciplinary approach. The GOM comprises basal metasediments, metabasalt slices, and schistose hornblende-bearing metagabbros, representing a tectonically imbricated, low-grade metamorphosed unit. Sentinel-2 satellite imagery, analysed using false colour composites (FCCs), principal component analysis (PCA), band ratios, and textural correlation, effectively discriminated lithological units and structural patterns. These results were validated and extended using high-resolution aeromagnetic data. Interpretation of aeromagnetic, employing advanced edge-detection filters such as the improved horizontal tilt derivative (impTDX) and STDR, together with classical techniques including the first vertical derivative (FVD), horizontal gradient magnitude (HGM), and tilt derivative (TDR), as well as three-dimensional (3D) Euler deconvolution and 3D magnetic modeling, revealed a network of NW–SE, NE–SW, N–S, and E–W trending faults at depths of 124–782 m. Within the SGC, NW-trending shear zones indicate late orogenic extensional exhumation associated with Najd fault-related tectonics. These structures govern the distribution of gold and radioactive (K, U, Th) mineralization. The results highlight the effectiveness of integrating remote sensing and aeromagnetic techniques for resolving lithological complexity, subsurface architecture, and mineral potential in structurally complex terranes worldwide.

**Supplementary Information:**

The online version contains supplementary material available at 10.1038/s41598-026-43824-x.

## Introduction

The Arabian-Nubian Shield (ANS), a key component of the East African Orogeny (EAO), spans a vast region encompassing the Eastern Desert of Egypt, southern Sinai, western Saudi Arabia, and northeastern Sudan^1–3^. Bounded by pre-Neoproterozoic crust on both its eastern and western margins^4^, the ANS represents one of the largest tracts of juvenile Neoproterozoic continental crust on Earth. Within Egypt, the Eastern Desert is traditionally subdivided into the Northern (NED), Central (CED), and Southern (SED) Eastern Deserts, each exhibiting distinctive lithotectonic record of Neoproterozoic magmatism, metamorphism, and deformation^5,6^.

The focus of this study lies within the SED, a structurally complex and mineral-rich segment of the ANS. The region records multiple phases of tectonic evolution, with assemblages ranging from ophiolitic remnants to arc-related granitoids, reflecting a prolonged geodynamic history. Abd El‑Wahed^7^ further subdivided the Egyptian ANS into three tectonic domains: the Northern Extensional Domain (NED), the Central Transpressional Domain (CTD), and the Southern Compressional Domain (SCD), each associated with distinct tectonothermal histories and mineralization patterns. This context highlights the importance of the Wadi Shait area as a representative segment for studying lithological and structural interactions in the SED. Such a geodynamically active setting offers an ideal setting for deploying integrated geological, geophysical, and remote sensing techniques aimed at unraveling the region’s mineral potential and tectonic history^8–11^. These rock assemblages form part of the ANS.

Integrating geological, geophysical, and remote sensing datasets presents a powerful, multidisciplinary approach for advancing mineral exploration and structural interpretation^12,13^. Geophysics tools play a crucial roles in mineral exploration^14,15^. One of the most reliable geophysical methods for defining subsurface structures is magnetics^16–35^ Aeromagnetic maps reveal anomalies i.e., variations in Earth’s magnetic field, that are useful for identifying specific geological units and aiding regional geological interpretation^36,37^. However, surface geological surveys alone often overlook critical subsurface features essential for comprehending the architecture and controls of mineralized systems. Therefore, integrating subsurface geophysical data with surface geological and remote sensing observations is imperative for a more comprehensive and accurate interpretation^6,38^.

In contemporary geological investigations, the synergistic application of remote sensing and geophysical data has emerged as a pivotal strategy for deciphering lithological and structural complexities, particularly in mineral exploration^12,39^. Remote sensing interpretations, when coupled with magnetic data, offer a powerful, cost-effective, and time-efficient approach to geological mapping. By leveraging the reflectance properties of specific spectral bands, which exhibit distinct responses based on mineral composition^40^, remote sensing provides a nuanced understanding of surface lithology. This, combined with the depth penetration capabilities of magnetic surveys that reveal subsurface structures, leads to a more comprehensive and accurate characterization of geological terrains. This integrated methodology not only expedites the mapping process but also enhances the precision of mineral target identification, leading to a more focused and successful exploration effort.

This clearly highlights the area’s pronounced lithological and structural complexity, as it comprises a diverse assemblage of geological units, including ophiolitic mélanges, island arc granitoids, metavolcanics, and metasedimentary rocks. This heterogeneity, overprinted by multiple phases of deformation and metamorphism, has made it a challenging region for geological interpretation. Although several structural studies have been undertaken to decipher the tectonic evolution of the Wadi Shait area^3,41–46^, these investigations were primarily based on field mapping, petrographic analysis, and conventional structural interpretation. While they significantly improved understanding of deformation phases and regional tectonics, they did not integrate high-resolution hyperspectral remote sensing, advanced aeromagnetic edge-detection filtering, three-dimensional magnetic modeling, or structural complexity analysis. Consequently, the subsurface structural architecture and its direct relationship to mineralization remain incompletely constrained. Given its position within the ANS and the imprint of key tectonic events, the study area is therefore considered to possess significant, yet under-evaluated, mineral potential.

Accordingly, this study investigates the lithological and structural complexity of the GOM and SGC in the Wadi Shait area through an integrated multidisciplinary approach. Despite the abundance of studies on the ANS, limited attention has been paid to combining hyperspectral remote sensing, enhanced magnetic filtering techniques, Euler depth solutions, and 3D magnetic modeling for mineral potential assessment in this locality. The central hypothesis driving this research is that integrating remote sensing, detailed aeromagnetic interpretation, and geochemical constraints can reveal previously unrecognized structural controls and lithological boundaries critical to mineralization. By applying high-resolution datasets and advanced edge-detection filters, this study provides new insights into both the geological evolution and mineral prospectivity of a key segment within the SED, offering a more robust framework for identifying structurally controlled mineralized zones.

## Geological and tectonic setting

**Fig. 1:**
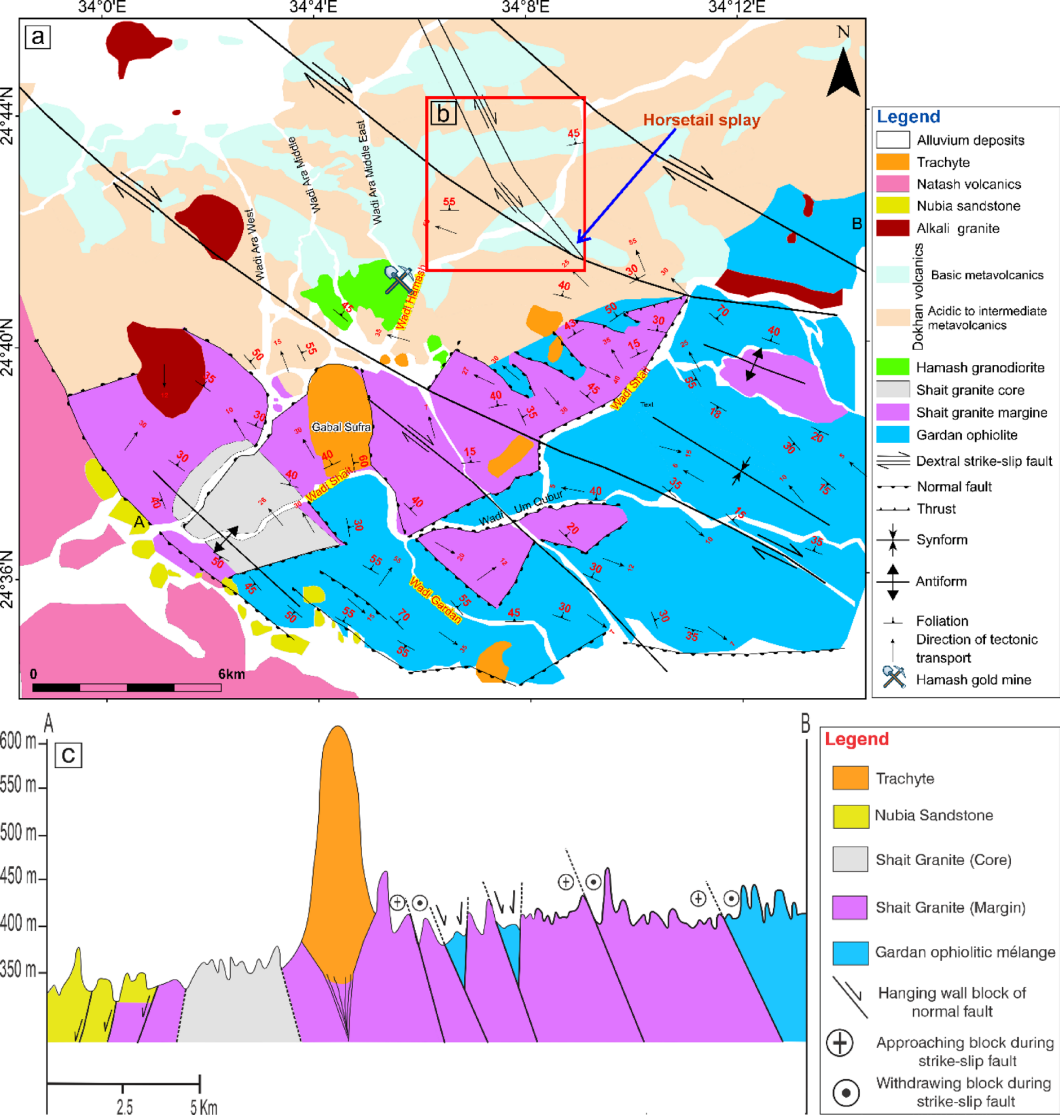
(**a**) Geological map of Wadi Shait area, modified after^3,41,42,45,47^. (**b**) Portion of the geological map highlighting a possible horsetail splay geometry developed at the termination of a dextral strike-slip fault. (**c**) Cross section of Wadi Shait area, modified after^3^. The figure was created by ArcGIS Desktop v 10.7.1. software; (https://www.esri.com/en-us/arcgis/products/arcgis-desktop/overview).

The Precambrian basement of the study area is tectonically bordered on the west by down-faulted blocks of Nubian Sandstone (Fig. 1). The Wadi Shait study area lies approximately 110 km east of Kom Ombo city in the Nile Valley. Spanning around 650 km^2^, this region is situated between latitudes 24° 33’ 00’’ to 24° 47’ 00’’ N and longitudes 33° 58’ 00’’ to 34° 14’ 00’’ E. El-Fadly, et al. ^41^ documented a tectonostratigraphic sequence within the basement rocks of the study area, starting with the GOM, which is intruded by the Shaitan granite complex (SGC), and displaying both brittle and ductile deformations. The SGC is overlain by the Hamash granodiorite, partially overprinted by several generations of faults. The sequence continues with the Dokhan volcanics, followed by post-orogenic alkaline granites and trachytes. The Nubian sandstones unconformably overlie these units and are locally intruded by Phanerozoic trachyte suites.

The GOM, traversed by Wadi Gardan to the west, comprises a deformed tectonic sequence organized into conformable nappes. The Eastern Desert ophiolites were emplaced during the final collision between East and West Gondwana (850–620 Ma), associated with the closure of the Mozambique Ocean^48^. A complete ophiolitic succession typically includes a mantle unit composed of serpentinized ultramafic rocks (peridotites) and a crustal unit consisting of gabbros (layered and isotropic), sheeted dykes, and massive or pillow basalts^49,50^. However, due to extensive folding and shearing, most Egyptian ophiolites are missing one or more of these characteristic lithologies^51^. The GOM forms a gray to dark-gray schistose terrain characterized by moderate to low relief, contrasting sharply with the elevated areas marked by Mesozoic trachyte and rhyolite plugs. From the base upward (or eastward), the sequence of GOM includes bedded metasediments, followed by metabasalt tectonic slices and schistose hornblende metagabbros^41^. The GOM exhibits well-defined contacts with the surrounding units of the SGC and Dokhan volcanics and is treated here as a distinct structural sequence.

Metamorphically, the GOM underwent initial greenschist-facies regional metamorphism, followed by contact metamorphism related to intrusion of the SGC, reaching hornblende-hornfels facies^52^. Subsequent retrogressive dynamic metamorphism resulted in pervasive shearing and diaphthoresis, transforming primary lithologies into protomylonites, mylonites, augen schists, and phyllonites.

The GOM occurs in two principal outcrops: a larger southern exposure along the margin of the SGC and a narrower east–west strip between the northern margin of the SGC and the southern Dokhan volcanics^41^. Both exposures display intense deformation and tectonic contacts with adjacent lithologic units, including intrusive contacts with granitoids and tectonic boundaries marked by strike-slip and normal faulting.

The SGC is a prominent feature in the Eastern Desert of Egypt, covering approximately 85 km^2^ and extending for about 20 km along the central part of the study area. Wadi Shait and Wadi Hamash, two major valleys, traverse the SGC and flow westward through the Nubian sandstone sequence^3^. The SGC rocks range from dark grey to light grey in colour, with occasional light red hues. Geochemical investigations of the SGC indicate that it belongs to a calc-alkaline, meta-aluminous granitoid suite characteristic of arc-related magmatism within the Arabian-Nubian Shield^3^. These granitoids are interpreted to have formed during the late stages of island-arc accretion and crustal thickening associated with the assembly of eastern and western Gondwana in the late Cryogenian–Ediacaran (~ 650 Ma). This interpretation is consistent with the juvenile arc-related evolution of the ANS and reinforces the volcanic arc affinity of the SGC. The SGC is bordered by the GOM to the south and northeast, the Dokhan volcanics and Hamash granodiorite to the north, and the Natash volcanics and Nubian Sandstone to the west.

The SGC intrudes the surrounding GOM, establishing a clear intrusive relationship between the two units. This contact reflects the emplacement of granitoid magmas into previously formed ophiolitic assemblages during the Late Tonian-Early Cryogenian tectonic evolution of the area. The contacts between the SGC and the Dokhan volcanics, Hamash granodiorite, and Natash volcanics are predominantly intrusive, although subsequent normal faulting has locally obscured these relationships. The contact between the SGC and the Nubian Sandstone is tectonic, marked by down-faulting of the SGC southwestward beneath the sandstone succession. The down-faulted block represents a portion of the western limb of a major antiform (Fig. 1) and is unconformably overlain by sandstone beds. The exhumation of the SGC is interpreted to be associated with the Najd shear zone, similar to structural patterns documented at Wadi Beitan^53^, El-Sibai^54–56^, and Wadi El-Shalul^57^. Furthermore, the SGC is intruded by several lens-shaped masses and dykes of Phanerozoic alkaline trachyte and rhyolite, with sharp and well-defined contacts.

The SGC is subdivided into two principal zones based on lithology and deformation: a core zone and an outer zone, separated by a gradational contact. The core zone, located in the western part of the complex, consists predominantly of mesocratic tonalite forming medium- to high-relief hills. The outer zone comprises light grey tonalite intruded by lens-shaped and dyke-like bodies of trondhjemite, granodiorite, and monzogranite. The outer zone is structurally more deformed than the core and is affected by NW-trending shear zones and fault systems. These structures locally overprint the granitoids and contribute to the complex structural framework of the area. The western part of the outer zone is dissected by high-angle normal faults, which resulted in subsidence and subsequent burial beneath the unconformable Nubian Sandstone succession (Fig. 1).

The Hamash granodiorite, a medium- to coarse-grained, dark green to greenish-grey rock, intrudes the SGC^41^. It is a late to post-orogenic granite intrusion^42,58^. The intrusion is locally characterized by spheroidal weathering and is dissected by numerous quartz veins and veinlets, reflecting hydrothermal activity associated with its emplacement. These veins commonly trend north–south and dip westward^59^. The Hamash granite is further intruded by dark-colored dolerite and pink rhyolite dikes related to the Natash Volcanics^60^. Structurally, the unit is affected by shearing, mylonitization, and NW–SE jointing^59^, contributing to its complex deformation history.

The Dokhan volcanics, which extruded over both the SGC and the Hamash granodiorite, cover the northern part of the study area. Although primary extrusive contacts are locally obscured by later faulting, xenolithic material derived from the SGC and Hamash granodiorite indicates magmatic interaction between these units^41^. The Dokhan volcanics consist predominantly of basaltic lava flows and pyroclastic deposits, conformably overlain by andesite lava and capped by felsic volcanic porphyry^60^. Structurally, the Dokhan volcanics are transected by NW-trending dextral strike-slip faults, consistent with regional structural trends.

The post-collisional Younger Granites^61,62^ occur as dyke-like masses in the northeastern and northwestern parts of the study area, intruding the SGC, GOM, and Dokhan volcanics. These intrusions reflect late-stage magmatic activity associated with post-orogenic tectonic evolution.

The tectonic evolution of the Wadi Shait area has revealed a complex polyphase tectonic history involving four main deformation events (D1–D4)^41^, each contributing to the region’s intricate structural framework. The earliest deformation phase (D1), dated to the Late Tonian–Early Cryogenian (~ 800 Ma), is related to the emplacement of the GOM and the intrusion of the SGC, interpreted as an immature ensimatic island arc granite^45^. This tectonic activity occurred within a back-arc basin of the Mozambique Ocean between fragments of the Rodinia Supercontinent^63^. The mélange experienced greenschist-facies marine metamorphism (M1), followed by contact metamorphism (M2) from the granitoid intrusion, and was subsequently overprinted by early NW-trending fabrics. The second deformation phase (D2), dated to ~ 630 Ma, involved NW-directed thrusting along NW-trending shear zones, producing mylonitic foliation striking NW and dipping 20–45° NW, with stretching lineations trending NW and plunging 2–40° NW along the Gardan mélange-SGC boundary during EAO^64^. The third phase (D3) produced a major antiformal structure with a NW-trending fold axis, similar to those at Meatiq, Hafafit, and El-Sibai, linked to late-orogenic exhumation and sinistral transpression along the Najd Fault System, foliation associated with this phase generally strikes NW-SE, dipping 10–70° NE on the eastern limb and 10–30° SW on the western limb, with NW–SE-trending stretching lineations plunging 5–45°^41,55,65^. Finally, the D4 phase introduced NW- and SW- trending brittle normal faults, which dislocated the western parts of the basement and were later concealed beneath Nubian Sandstone^66,67^.

## Materials and methods

**Fig. 2:**
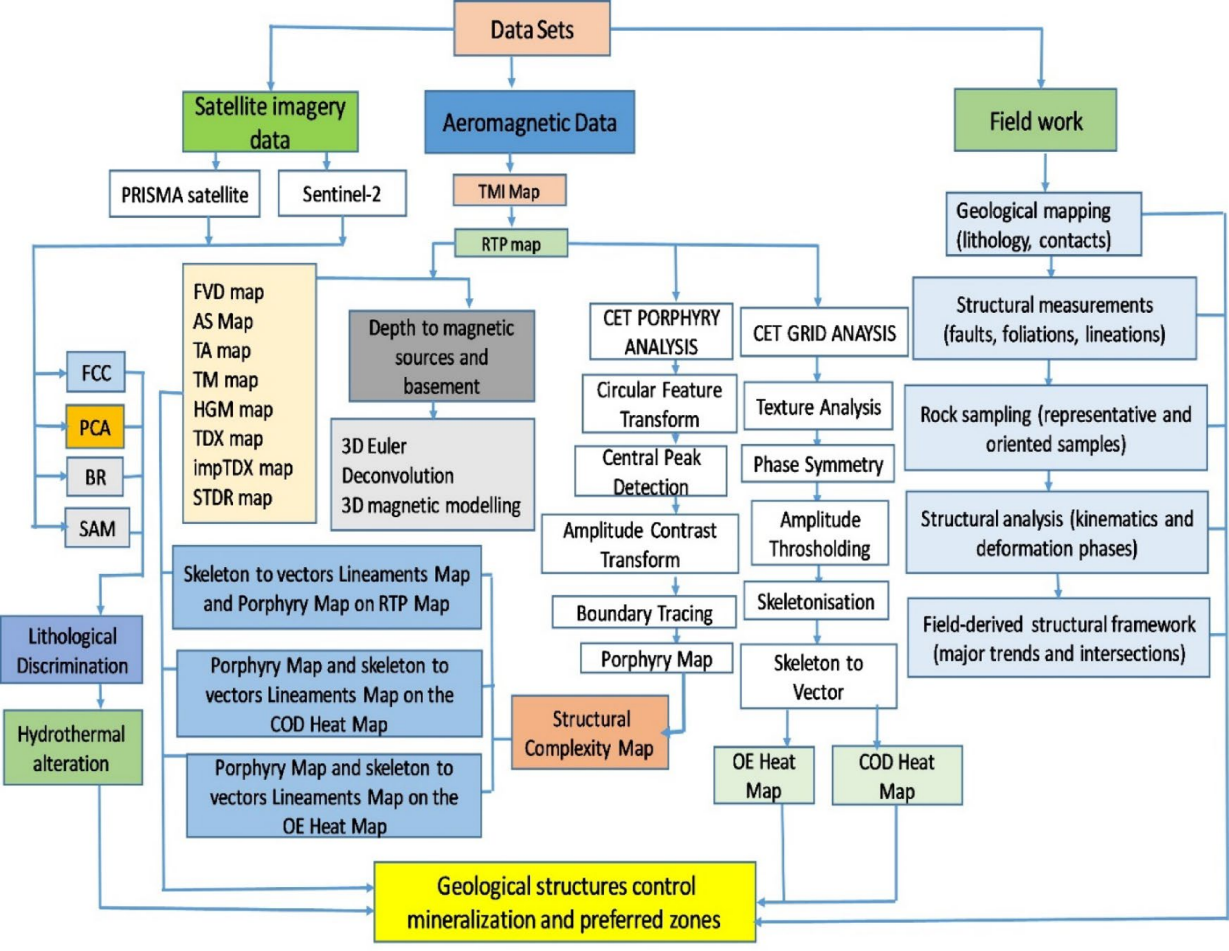
Flowchart showing the methodology used in the present study.

Figure 2 summarizes the methods for this study, integrated workflow illustrating the processing and interpretation of satellite imagery, aeromagnetic data, and field-based geological observations. Field work provides geological mapping, structural measurements, sampling, and structural analysis that define a field-derived structural framework, which is integrated with remote sensing and aeromagnetic results to identify structural controls on mineralization and preferred exploration zones.

### Remote sensing data and applied techniques

The current contribution’s main aim is the lithological discrimination of outcropping rock units, hydrothermal alteration patterns, structural elements, implementing field data with specific remote sensing techniques. To achieve these objectives, Sentinel-2 multispectral imagery was obtained from the Copernicus Data Space platform (https://dataspace.copernicus.eu), providing spectral coverage across the visible, near-infrared, and shortwave infrared regions. To advance beyond the capabilities of multispectral analysis, this study also integrated hyperspectral data from PRISMA (PRecursore IperSpettrale della Missione Applicativa). PRISMA offers 242 contiguous spectral bands spanning the visible to shortwave infrared ranges, allowing for a more precise characterization of the spatial distribution of hydroxyl-bearing minerals and iron oxides within the investigated area.

The FCCs, PCA, band ratios, spectral angle mapper, and simplified textural analysis through correlation component^68^ were employed. FCCs are composite images formed by three spectral ranges in RGB, designed to visually enhance the discrimination of rock units. Although simple, FCCs are widely recognized as a reliable tool for lithological discrimination in various studies^12,69,70^. The PCA, a multivariate statistical method for multispectral image interpretation, was employed to decorrelate data and reduce the dimensionality^12,71–73^. By reorienting orthogonal axes at the data mean, PCA constructs uncorrelated linear combinations of variables (eigenvector loadings) to optimize variance distribution. Although the terminal bands dominated by noise appear less significant, the output PCA bands correspond to the input spectral bands, with the foremost band encapsulating the maximum variance and subsequent bands capturing progressively diminished proportions. Band ratio, as the name implies, is a digital image processing method widely used in hydrothermal alteration mapping^40,47,74,75^ through calculating the ratio of a pixel’s reflectance in one spectral band to its corresponding pixel in another band, thereby enhancing the spectral contrast between electromagnetic bands. Spectral Angle Mapper (SAM) assigns a weighting coefficient based on the spectral similarity between each pixel in the image and the corresponding end member for each class^76,77^.

### Aeromagnetic data and processing

The aeromagnetic data collection employed belongs to the Egyptian geological survey maps at the scale of 1:50,000 and was collected in 1983–1984 by the General Petroleum Company of Egypt (EGPC) with assistance from the American Aero Service Division of the Western Geophysical Company. The traverse lines go from north to east at a 45° angle and are separated by about 1.0 km. Perpendicular to the traverse lines, the tie lines are about 10 km apart and run in the direction of N 135° E.^78^. The survey was conducted with an azimuth of 45° and 225° from true north, following parallel flight lines in the NE-SW direction at 1.5 km intervals at a notional terrain clearance of 120 m. The Geosoft Oasis Montaj V. 8.4 was used for data gridding, processing, and visualization. The UTM coordinate system with zone 36 N was used to project the magnetic data. After deducting the International Geomagnetic Reference Field (IGRF), the total magnetic intensity (TMI) data was gridded and transformed to the frequency domain using Fast Fourier Transform (FFT). Reduction parameters of 34.45˚N inclination and a 1.93˚E declination, were used for data reduction to the north magnetic pole in order to reposition the magnetic anomalies directly above their causative sources^79,80^. The resulting reduced to the pole (RTP) magnetic data subjected to several enhancing techniques to improve readability for interpretation as follows:

#### First vertical derivative (FVD)

Nabighian^81^developed FVD technique, which uses 3D Hilbert transformations in the axes of x and y. In this work, shallow features with their borders and related lineaments are enhanced by their utilization. While attenuating long-wavelength components, this filter enhances short-wavelength anomalies, thereby emphasizing structural discontinuities and revealing anomaly textures through zero-contour lines^82,83^.

#### Horizontal gradient magnitude (HGM)

Possibly the simplest technique for locating magnetic contact at depths is Blakely and Simpson^84^ HGM technique, which only considers the domain’s two first-order horizontal derivatives rather than vertical derivatives. The HGM map is one of the mathematical methods for analyzing and comprehension magnetic data^85^. Therefore, for a grid with a magnetic field value of T(x, y), the HGM is stated as follows:

1 $${\mathrm{HGM}} = \sqrt {\left( {\frac{{\partial F}}{{\partial X}}} \right)^{2} + \left( {\frac{{\partial F}}{{\partial Y}}} \right)^{2} }$$

According to Pilkington and Boulanger^86^, the HGM method offers the highest resolution among all edge detection techniques. A key limitation of HGM, however, is its sensitivity to the direction of magnetization. This drawback can be mitigated by applying RTP transformation to the original magnetic data. Within the study area, the HGM technique was applied to RTP-transformed data to delineate the positions and structural trends of magnetic source bodies.

#### Tilt angle (TA)

The TA is determined by Eq. (2), which gives the ratio’s arctangent of the magnetic field’s vertical to horizontal derivatives^87^.

2 $${\text{TA }} = {\text{ tan}}^{{ - {\mathrm{1}}}} \left( {{\mathrm{VDR}}/{\mathrm{HGM}}} \right)$$

Specifically, TA is used here to strengthen the edges of fault lineaments and magnetic sources. The TA ranges from − π/2 to + π/2, with values near zero typically occurring above the edges of causative bodies, while high positive values are generally centered over the magnetic sources.

#### Theta map (TM)

The ratio between the magnitudes of AS and the total horizontal derivative is known as cos (θ). Because it effectively balances anomalies with high and low amplitudes and potentially highlights borders of various sizes of hidden geological structures, this technique has been extensively employed^88^. The magnetic sources’ locations in the region are determined by the theta map’s maximum value. It is calculated using the following equation:

3 $${\mathrm{TM}} = \frac{{\sqrt {\left( {\frac{{\partial F}}{{\partial x}}} \right)^{2} + \left( {\frac{{\partial F}}{{\partial y}}} \right)^{2} } }}{{\sqrt {\left( {\frac{{\partial F}}{{\partial x}}} \right)^{2} + \left( {\frac{{\partial F}}{{\partial y}}} \right)^{2} + \left( {\frac{{\partial F}}{{\partial z}}} \right)^{2} } }}$$

#### Horizontal tilt angle (TDX) and improved TDX (impTDX)

Cooper and Cowan^89^ introduced the TDX, which is defined as the arctangent of the ratio between the horizontal derivative amplitude and the absolute value of the vertical derivative. It is expressed as:

4 $${\mathrm{TDX}} = {\mathrm{atan}}\left( {\frac{{\sqrt {\left( {\frac{{\partial F}}{{\partial x}}} \right)^{2} + \left( {\frac{{\partial F}}{{\partial y}}} \right)^{2} } }}{{\left| {\frac{{\partial F}}{{\partial z}}} \right|}}} \right)$$

The TDX filter is highly effective in enhancing gradients over source bodies, offering strong edge sharpening capabilities. However, it tends to exaggerate boundary widths, particularly for deep and intermediate-depth structures, which may lead to less precise delineation of their edges. The impTDX filter is an improved version of the TDX filter that has been introduced by Ibraheem, et al. ^90^ to address this issue and provide several other benefits. It is a unique edge detector utilizing the hyperbolic tangent function. This technique is applied after reducing the magnetic data to the north magnetic pole. It is given using the following equation:

5 $${\mathrm{impTDX}} = {\mathrm{atan}}\left( {M.\frac{{\left( {\frac{{\partial ^{2} F}}{{\partial Z^{2} }}} \right)}}{{\sqrt {\left( {\frac{{\partial TDX}}{{\partial x}}} \right)^{2} + \left( {\frac{{\partial TDX}}{{\partial y}}} \right)^{2} } }}} \right)$$

where M is the average magnetic field strength in the research region, and the $\frac{{\partial ^{2} F}}{{\partial Z^{2} }}~$is the second vertical derivative of the observed magnetic field. However, applying the second vertical derivative may amplify noise in the data. Therefore, using the Laplace equation, the second-order horizontal derivatives can be substituted with the vertical derivative. Accordingly, Eq. (6) can be reformulated as follows:

6 $${\mathrm{impTDX}} = \tanh \left( {\frac{{ - M.~\left( {\frac{{\partial ^{2} F}}{{\partial X^{2} }} + \frac{{\partial ^{2} F}}{{\partial y^{2} }}} \right)}}{{\sqrt {\left( {\frac{{\partial TDX}}{{\partial x}}} \right)^{2} + \left( {\frac{{\partial TDX}}{{\partial y}}} \right)^{2} } }}} \right)$$

Due to the characteristics of the hyperbolic tangent function and the peak response of the new filter across the entire causative body, the impTDX transform ranges from − 1 to + 1. This filter effectively enhances the edges of both shallow and deep structures and accurately delineates adjacent magnetic sources. Moreover, it balances the signal amplitudes from sources at varying depths, ensuring consistent edge detection. To further improve edge clarity, the horizontal derivative of impTDX (THDR_impTDX) is calculated, which emphasizes maximum values along the boundaries of magnetic bodies^90^. This is determined using Eq. (7).

7 $${\mathrm{THDR}}\_{\mathrm{impTDX}} = \sqrt {\left( {\frac{{\partial {\mathrm{impTDX}}}}{{\partial x}}} \right)^{2} + \left( {\frac{{\partial {\mathrm{impTDX}}}}{{\partial y}}} \right)^{2} }$$

#### STDR filter

This method delineates anomaly edges with high precision, clarity, and sharpness by integrating the intensity of both weak and strong anomalies, as well as those occurring at different depths^91^. It effectively separates adjacent causative bodies, addresses the diffusion problem commonly associated with edge detection, and enhances edge resolution. The filter also characterizes both positive and negative anomalies based on their extreme values. According to Nasuti, et al. ^91^, this filter can be computed using the following equation:

8 $${\mathrm{STDR}} = {\mathrm{tan}}^{{ - {\mathrm{1}}}} \left( {M\frac{{\left( {\frac{{\partial ^{2} F}}{{\partial Z^{2} }}} \right)}}{{\sqrt {\left( {\frac{{\partial THD}}{{\partial x}}} \right)^{2} + \left( {\frac{{\partial THD}}{{\partial y}}} \right)^{2} } }}} \right)$$

Additionally, the total horizontal derivative of STDR was presented as follows to enhance the detection of source edges:

9 $${\mathrm{THDR}}\_{\mathrm{STDR}} = \sqrt {\left( {\frac{{\partial STDR}}{{\partial x}}} \right)^{2} + \left( {\frac{{\partial STDR}}{{\partial y}}} \right)^{2} }$$

The edges of the anomalies for both positive and negative anomalies are displayed by the maximum values of the THDR_STDR filter. However, it is worth mentioning that both the STDR and its THDR_STDR exhibit significant sensitivity to noise^92^.

#### Euler deconvolution (ED)

3D ED is a technique for identifying the location, profundity, and type of any sources present in data from gridded potential fields^93^. It is computed using Eq. (10):

10 $$X\frac{{\partial F}}{{\partial x}} + Y\frac{{\partial F}}{{\partial x}} + Z\frac{{\partial F}}{{\partial x}} + ~\eta F~ = X_{0} \frac{{\partial F}}{{\partial x}} + Y_{0} \frac{{\partial F}}{{\partial x}} + Z_{0} \frac{{\partial F}}{{\partial x}} + ~\eta b~$$

The data’s x-, y-, and z-derivatives are also a parameter known as the structural index (SI). According to Stavrev and Reid^94^, the SI, an integer value associated with the potential field’s homogeneity, changes depending on the type and the geometry of the source. In the context of total-field magnetic anomalies, an SI value of 3 typically corresponds to a sphere, an SI of 1 represents a dike, and an SI of 0 indicates a contact. These values are also used to cluster anomaly responses, helping to constrain the overall geometry of the source model.

#### Centre for exploration targeting grid analysis (CET)

The CET Grid Analyses method uses standard deviation to identify deposit occurrence favourability by analyzing an image’s texture to identify any areas of structural complexity and any laterally continuous line-like discontinuity, such as lineaments along ridges and edges. The CET technique is used to define exploration target regions and assess textures, lineations, and structural complexity. It is employed to delineate exploration target areas by evaluating magnetic textures, lineaments, and structural complexity. It combines bilateral symmetry detection with texture analysis to identify discontinuities in magnetic susceptibility^95^. Structural complexity analysis plays a key role in identifying favorable zones for ore deposits^96^. The CET approach detects structural features such as lineament intersections, junctions, and strike changes by emphasizing discontinuities and analyzing their spatial relationships. Lineament extraction is achieved through a three-step process: texture analysis, texture ridge identification, and texture ridge thinning.

#### 3D magnetic modelling

Enhanced geologic interpretation is possible via the integrated processing and interpretation of magnetic data made possible by GMSYS-3D of the Geosoft Inc. ^97^. On an interactive graphics interface connected to a host computer, 3-D model bodies are created from polyhedra with the appropriate geometry and physical properties (density and susceptibility). In an interactive mode, the approach is specifically designed for simultaneous processing and interpretation. Topographic reduction calculations vividly demonstrate the evolution of magnetic modeling approaches. This development started with manual approaches such as templates^98,99^. When utilizing pole-reduced magnetic data in magnetic model computations, the Poisson theorem will be employed^100^. It is often phrased as follows:

11 $$\frac{{\partial V}}{{\partial S}} = \frac{I}{{G~\left( {\frac{{\rho \partial ^{2} U}}{{\partial S\partial ~\theta }}} \right)}}$$

In this equation, V denotes the magnetic potential, U represents the gravity potential, I is the homogeneous magnetization of the body, G is the gravitational constant, ρ is the homogeneous density, θ indicates the direction of magnetization, and s denotes arbitrary differentiation.

The 3D magnetic inversion in in the current investigation was performed using the GYMSYS-3D tool within Oasis Montaj 2007. The modeling process was conducted in the wavenumber domain using Parker’s method^101^. The model assumes average values of magnetic susceptibility and remanent magnetization for each subsurface layer. An elevation grid and a corresponding basement rock grid were used to define the model geometry. Magnetic data were extracted from the RTP-transformed grid at an observation height of 1205 m. The magnetic susceptibility of the basement layer was set at 0.125663 SI unit (equivalent to 0.01 cgs).

### Field investigation and structural analysis

The field study was designed to achieve a comprehensive understanding of the geological framework and structural evolution of the study area. It involved detailed geological mapping, integrating remote sensing techniques using satellite imagery of Sentinel 2 and PRISMA data. These datasets facilitated the identification of lithological variations, major structural lineaments, and tectonic features prior to field verification.

Systematic field investigations were conducted to precisely delineate geological contacts and document macro- and meso-structural features, with particular emphasis on those associated with regional fault zones and deformational histories. Detailed structural measurements, including orientation data for folds, faults, and foliations, were recorded in the field to support subsequent kinematic and tectonic interpretations. Photographs documenting key field observations and structural relationships were also utilized to support the analysis. Approximately 400 rock samples were collected from 139 representative field sites distributed across the study area. The spatial distribution of these sampling sites is shown in Supplementary Figure S1. Representative-oriented samples were also extracted from critical profiles that intersected major exposed rock units to preserve structural information for further microscopic analysis. The integration of field observations and remote sensing data ensures a robust methodological approach to understanding the geological and structural evolution of the study area.

## Results and discussion

### Lithological mapping

**Fig. 3:**
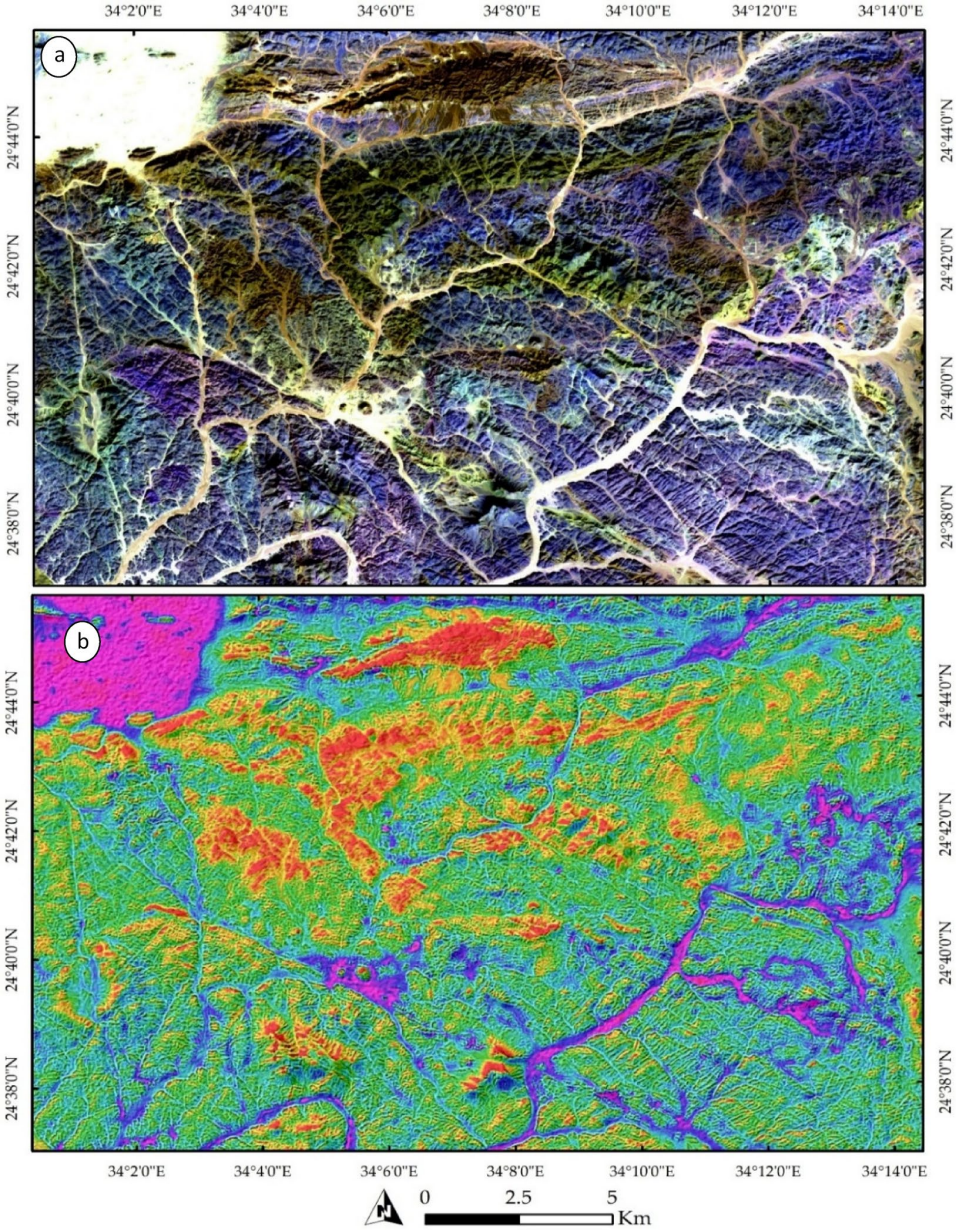
Lithological discrimination using Sentinel-2 imagery: (**a**) a FCC of bands 3, 6, and 12 displayed in RGB, respectively, and (b) a synthetic image derived from band 11, highlighting the distribution of acidic to intermediate metavolcanics and granitic rocks (shown in red) within the extensive ophiolitic mélange and basic metavolcanic units. The Magenta color indicates granitic rocks or their weathered products, which are sometimes deposited in wadis. The data were obtained from ESA, and the figure was created using ENVI v. 5.6.2 software; (https://www.l3harrisgeospatial.com/Software-Technology/ENVI ).

Our remote sensing analysis revealed that the study area exhibits a high-complexity lithological pattern. Various technique combinations and image processing were employed to differentiate the lithological features. A FCC of bands 3, 6, and 12 in RGB (Fig. 3a) effectively highlighted the widely distributed rock units in the study area, represented by a violet-colored matrix. This matrix primarily corresponds to the Gardan ophiolite (mainly in the southern part) and intermediate-to-basic metavolcanics (mainly in the northern part).

Other rock units were distinguishable inside this widespread lithological feature. For example, acidic metavolcanics appeared as dark-colored pixels, predominantly located in the northern part of the study area. Granitic rocks were identified based on their tone or form. Specifically, alkali granite bodies were characterized by their distinctive, prominent shapes, primarily in the western part of the study area. In contrast, the Hamash granodiorite, with a slightly different tone, was identified in the central region. Additionally, trachyte plugs were separated based on their elevation and texture, appearing as two main plugs concentrated in the central part of the study area.

**Fig. 4:**
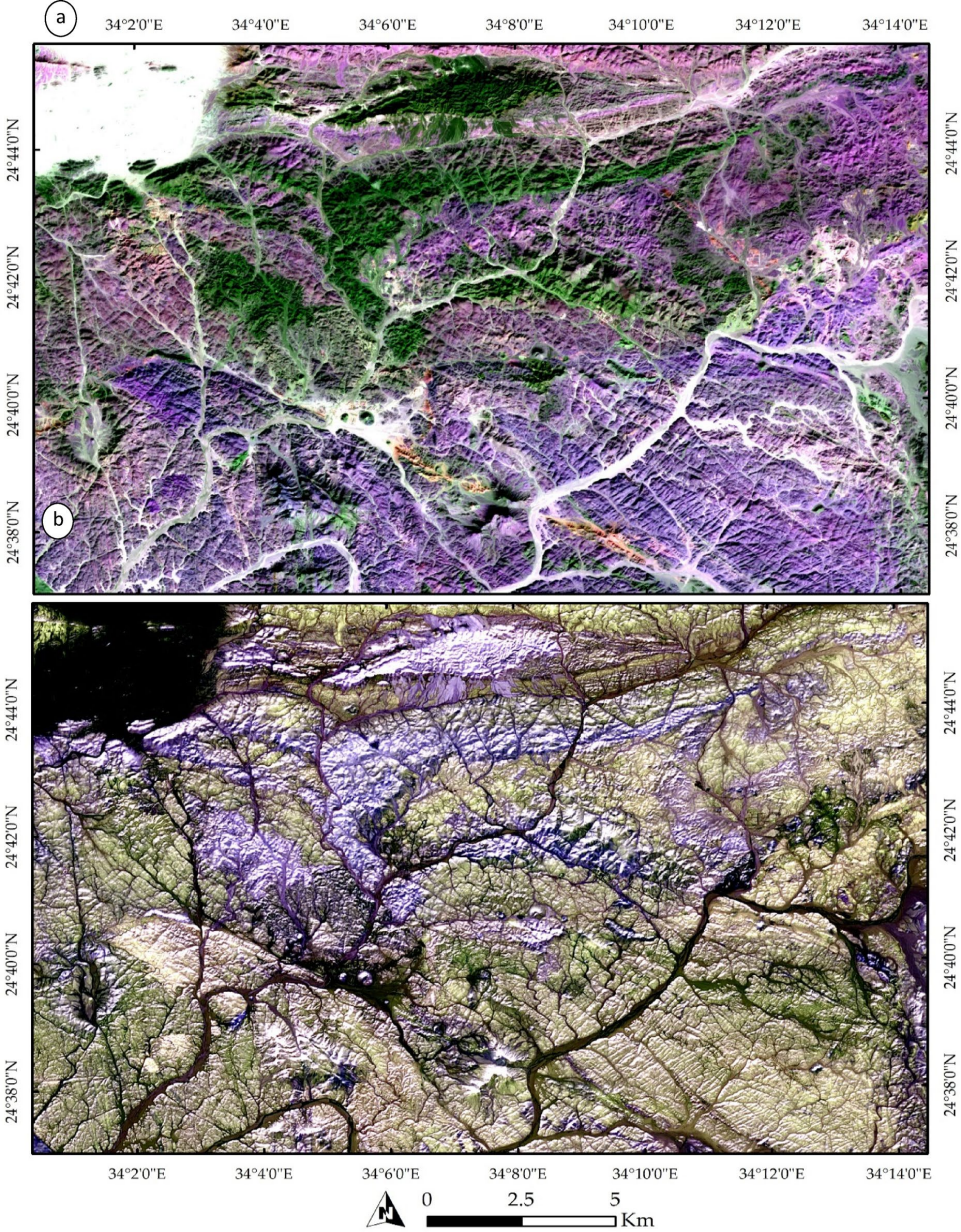
(**a**) Lithological discrimination using Sentinel-2 FCC of bands 11, 8, and 12 in RGB, respectively, and (**b**) lithological and structural delineation using Spectral Angle Mapper (SAM) rule images numbered 4, 6, and 3 in RGB, respectively. Note the clear depiction of the drainage patterns and their alignment with structural elements. The data was obtained from ESA, and the figure was created by ENVI v. 5.6.2. software; (https://www.l3harrisgeospatial.com/Software-Technology/ENVI.

A distinct distribution of all the rock bodies mentioned above is illustrated in Fig. 3b and marked in red. These bodies primarily represent the acidic metavolcanics, alkali granites, Hamash granodiorite, and trachyte plugs. A more detailed representation is provided in Fig. 4a, which clearly delineates the Shait granitic body, extending from east to west and appearing as a slight violet hue. The granitic margin is distinctly highlighted in green in Fig. 4b through SAM rule images using the 4-6-3 RGB combination.

**Fig. 5:**
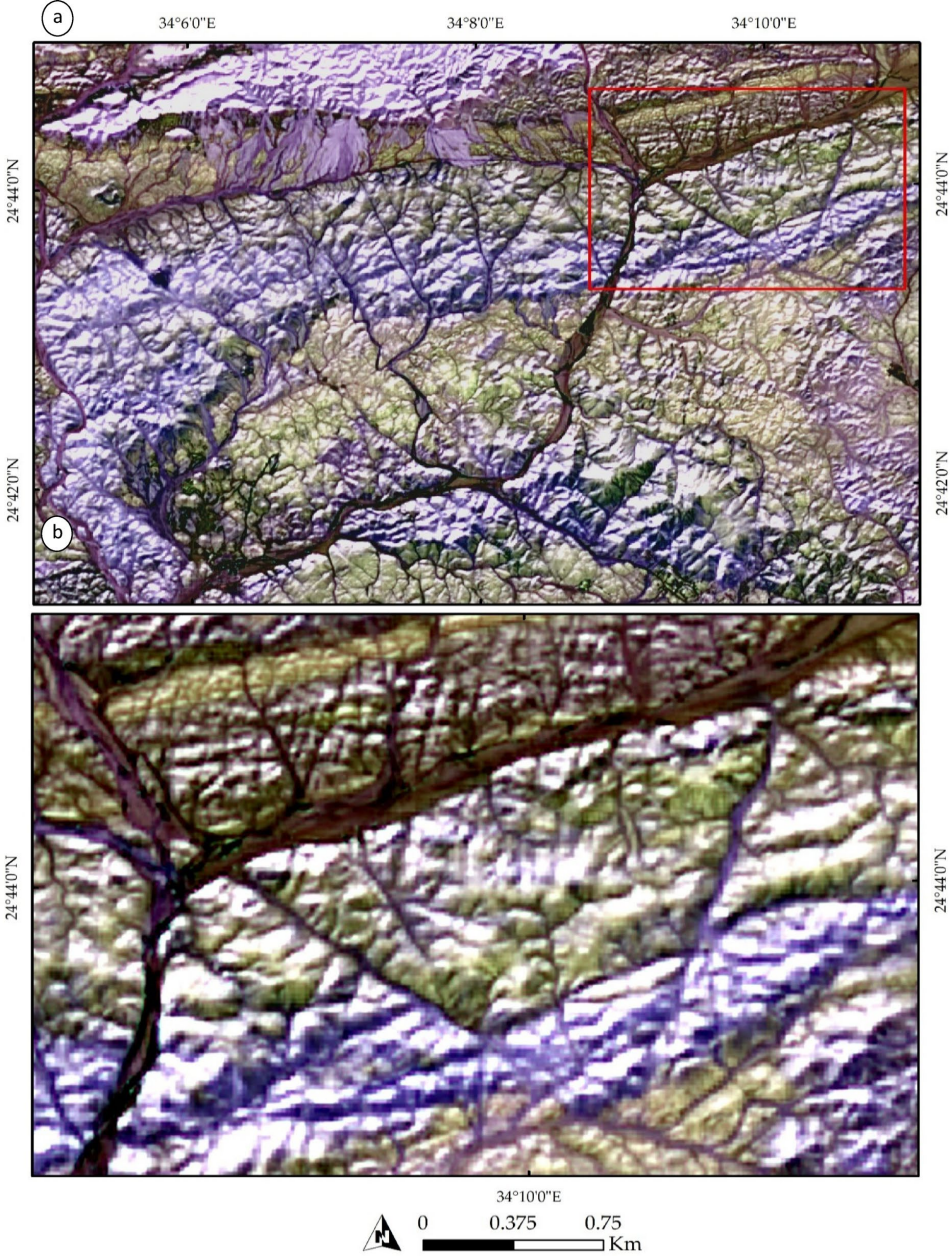
(**a**) Close-up view of the northeastern sector of the study area showing acidic metavolcanics dissected by NW–SE-trending faults. (**b**) Zoomed-in view of the boxed area in (**a**), illustrating the branching of subsidiary faults near the termination of a NW–SE-trending fault system, forming a geometry consistent with a possible horsetail splay. The data were obtained from ESA, and the figure was created using ENVI v. 5.6.2 software (https://www.l3harrisgeospatial.com/Software-Technology/ENVI).

Figure 5b also effectively emphasizes the primary structural trends within the study area, as indicated by the structurally controlled wadis. A comprehensive analysis of Fig. 4b reveals a dominant NW, NE, and NS orientation of wadis, reflecting the highly deformed nature of the studied terrain. A closer examination of these structural elements is provided in Fig. 5, where NNW-trending faults are shown displacing the metavolcanics. These faults are identifiable by tracing the trajectories of the wadis within the terrain as documented by our field observations and previous studies.

**Fig. 6:**
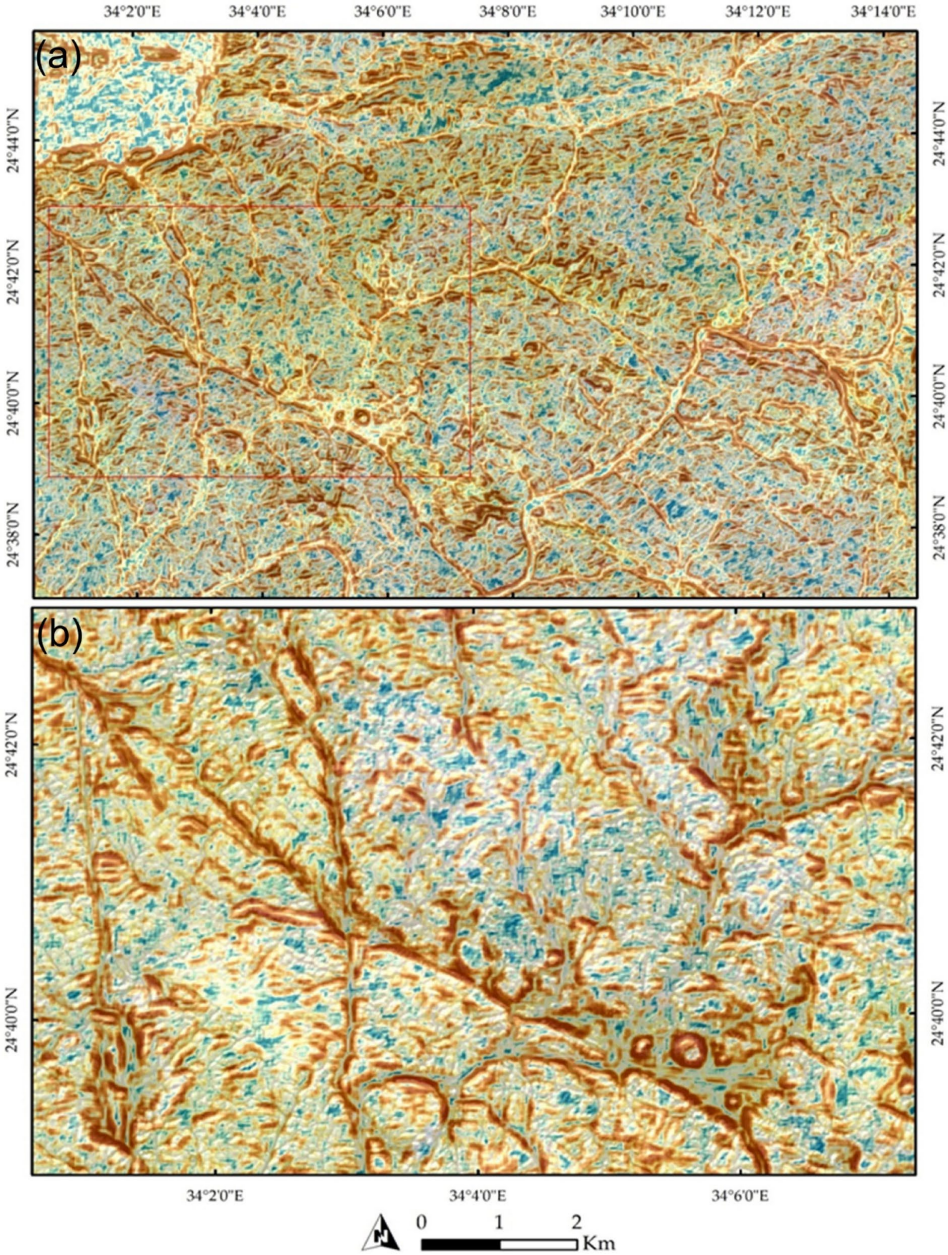
(**a**) A pseudo-color map depicting the correlation values of Sentinel-2 band 12 data, generated using a moving window size of 11 × 11 pixels, highlighting tectonic foliation trending approximately E-W in the northern part of the study area. (**b**) The map also reveals additional structural elements influenced by the drainage pattern and their intersections. The data was obtained from ESA, and the figure was created by ENVI v. 5.6.2. software; (https://www.l3harrisgeospatial.com/Software-Technology/ENVI).

A zoomed-in view of the northeastern part of the terrain is presented in Fig. 5a, illustrating the influence of NW- and NE-trending faults. The dextral strike-slip fault system appears to exhibit a horsetail splay geometry, inferred from the fan-like divergence of subsidiary lineaments near the termination of the main fault. This interpretation is supported by the systematic branching of lineaments and drainage alignments, which suggest localized distributed brittle deformation consistent with strike-slip fault propagation. This is further exemplified in Fig. 4b, where several wadies’ intersections are visible, providing additional evidence of the faulting patterns. These structural features are further corroborated by Fig. 6, which displays the correlation component of Band 12. This picture highlights the primary tectonic features, such as the E-W-trending foliations in the northern part of the study area, showcasing the interconnected wadis in brown, the principal tectonic framework. Additionally, a zoomed-in view of the main structural trends is provided in Fig. 6b.

**Fig. 7:**
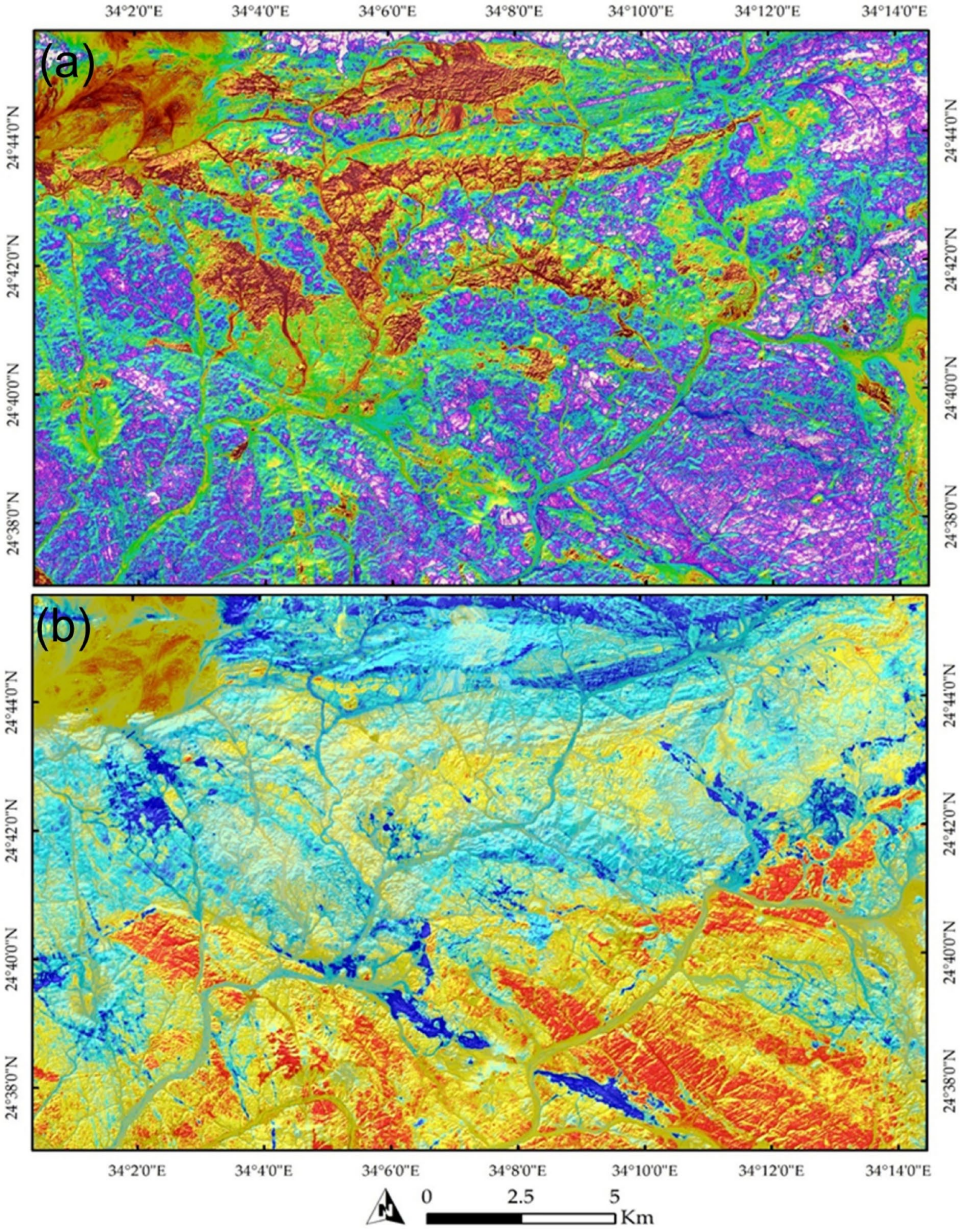
(**a**) A pseudo-color map of PC5 highlighting the distribution of granitic, granodioritic, and trachytic rocks in green. (**b**) A map showing a clear distinction between the northern part, dominated by metavolcanics (primarily shown in cyan), and the southern part, characterized by ophiolitic mélange (depicted in yellowish-red), with an emphasis on structural trends and highly tectonized zones marked in blue. The data were obtained from ESA, and the figure was created using ENVI v. 5.6.2 software (https://www.l3harrisgeospatial.com/Software-Technology/ENVI ).

The PCA results were instrumental in highlighting both the lithological and structural characteristics of the study area. Figure 7a illustrates PC5, effectively separating acidic metavolcanics, represented in brown and granitic compositions, shown as green-colored pixels (e.g., Hamash granodiorite and alkali granite at the western part). Figure 7b delineates the northern and southern parts of the study area using two distinct colors, reflecting variations in lithological facies between these regions. Additionally, it highlights prominent NW-trending structurally controlled zones, depicted in blue, which underscores the local structural control.

**Fig. 8:**
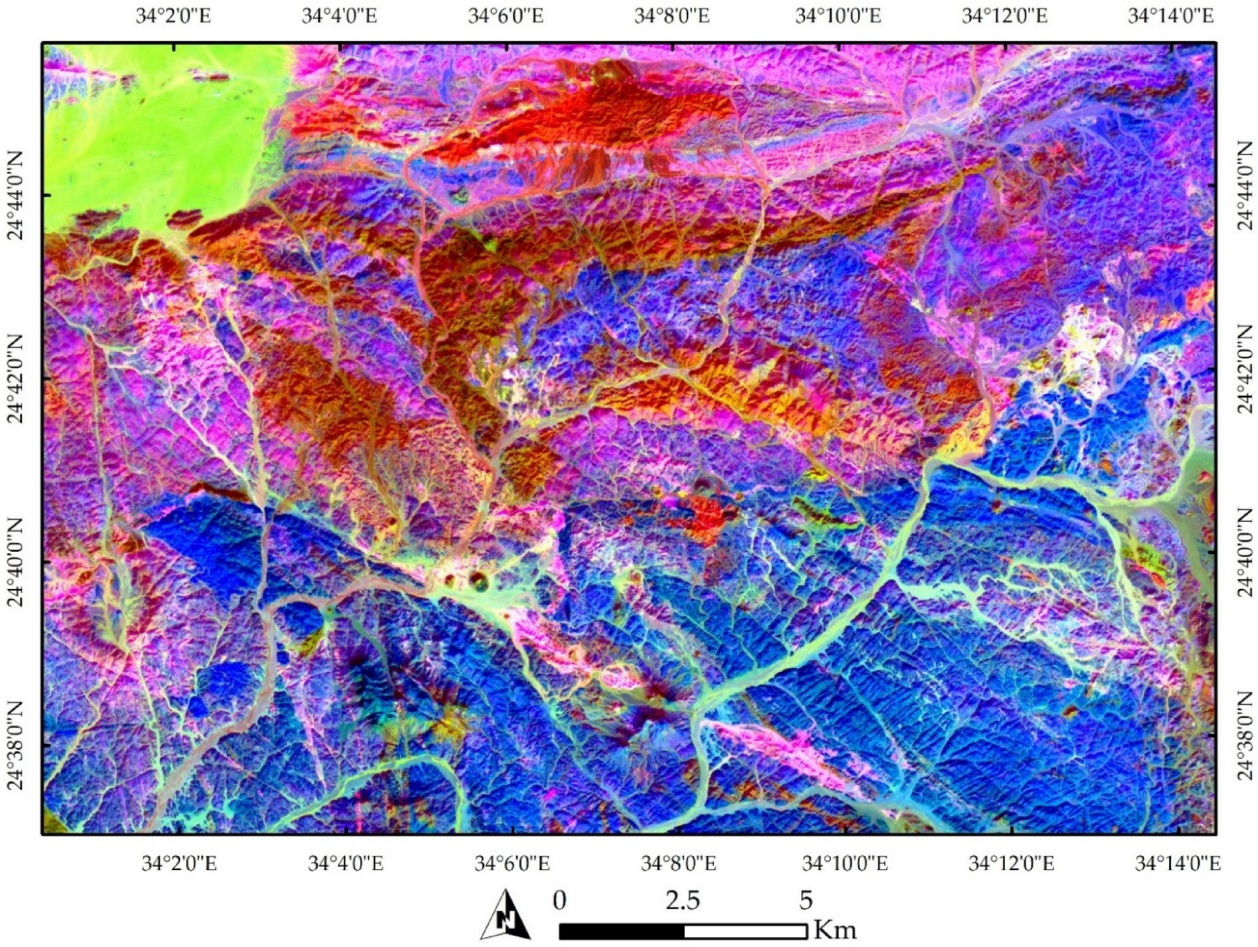
Visualization of general hydrothermal alterations using Sentinel-2 band ratio combinations (11/12, 4/2, and 11/8) displayed in RGB, respectively. The map highlights rocks enriched with hydroxyl-bearing minerals in pinkish to reddish hues, while rocks containing iron oxides are represented in blue and violet tones. The data was obtained from ESA, and the figure was created by ENVI v. 5.6.2. software; (https://www.l3harrisgeospatial.com/Software-Technology/ENVI ).

These structural elements are considered pathways for hydrothermal fluids, which play a crucial role in forming orogenic gold deposits within the Nubian Shield. Consequently, a preliminary detection of hydrothermal alterations was conducted using the widely acknowledged Sabins band ratio^40,102^, as shown in Fig. 8. In this figure, hydroxyl-bearing minerals are depicted in a pinkish color, primarily associated with NW-trending rocks and granitic formations within the study area. These granitic rocks are well-known for hosting gold mineralization. Conversely, the blue pixels represent lithologies containing Fe-bearing minerals.

**Fig. 9:**
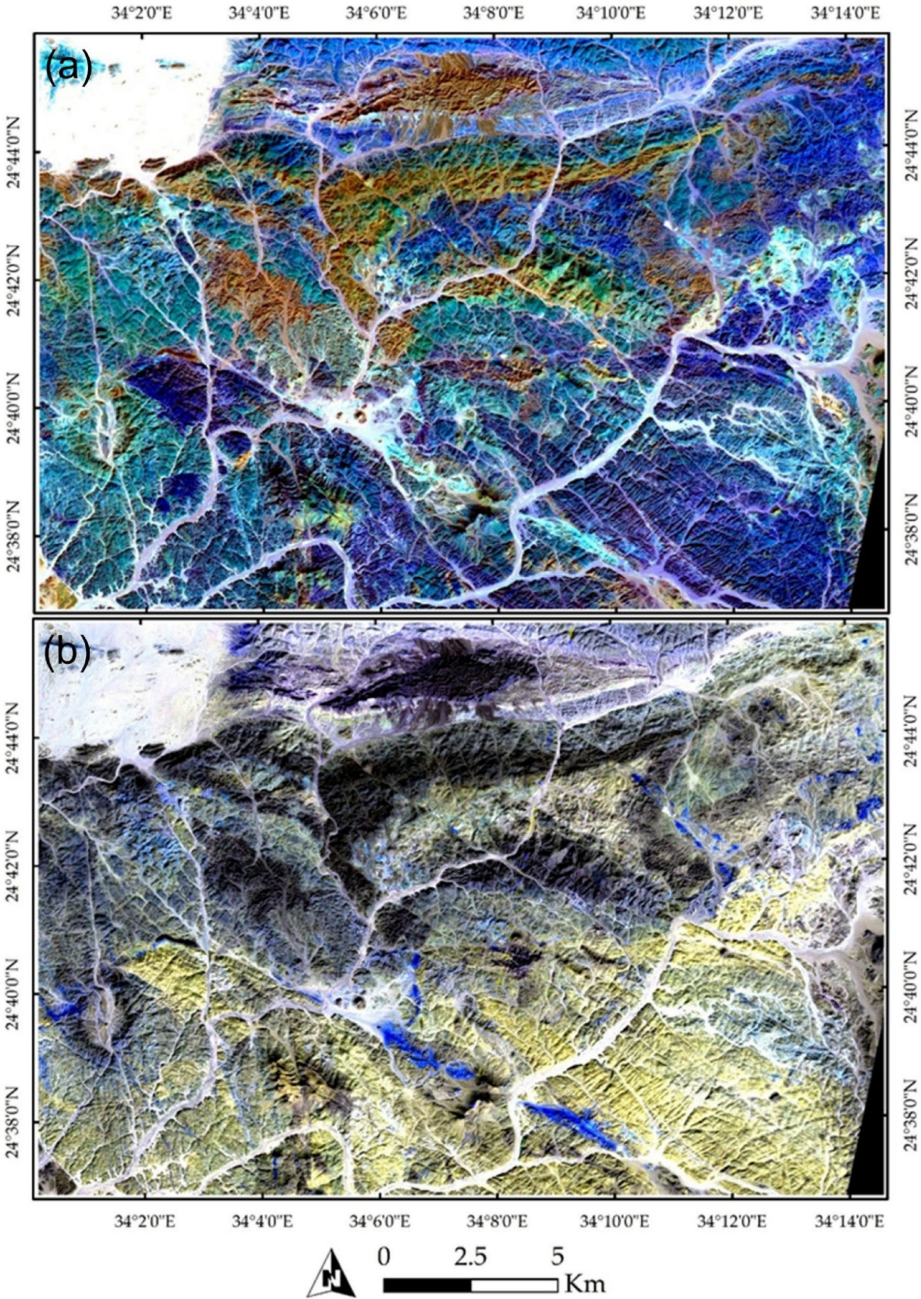
FCCs derived from PRISMA hyperspectral data, highlighting altered rock units within the study area in (**a**) Altered zones displayed in cyan and (**b**) Altered zones represented in vivid blue.

**Fig. 10:**
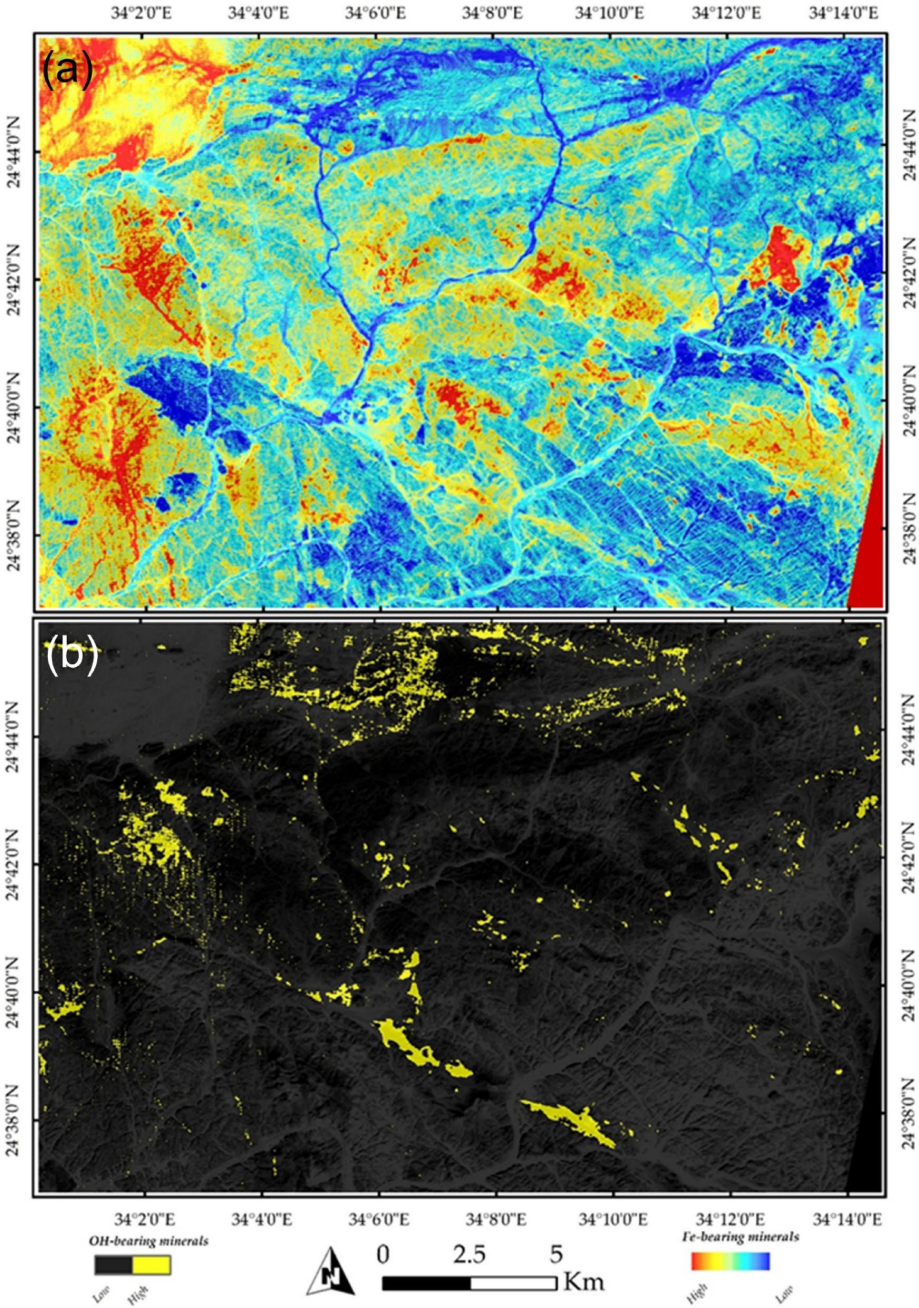
Band ratio maps from PRISMA data hydrothermal alteration types. (**a**) Iron oxides identified using the VNIR ratio (∼664 nm / 497 nm), shown in yellow to red tones. (**b**) OH-bearing minerals mapped using the SWIR ratio (∼1596 nm / 2442 nm), displayed in yellow hues.

The analysis of PRISMA hyperspectral data provided significant improvements over the lithological complexity initially interpreted from Sentinel-2 imagery. PRISMA was specifically employed to enhance the detection and mapping of hydrothermal alteration zones. For example, Fig. 9 illustrates a FCC, where altered rock units are distinctly highlighted—appearing in cyan in Fig. 9a and in vivid blue in Fig. 9b. Furthermore, targeted band ratio techniques were applied to isolate specific mineralogical features. A ratio of VNIR bands (∼664 nm / 497 nm) effectively distinguished iron oxides, which appear in yellow to red tones in Fig. 10a. Similarly, the ratio of SWIR bands (∼1596 nm / 2442 nm) clearly emphasized the distribution of OH-bearing minerals, represented in yellow hues in Fig. 10b. When combined, these spectral enhancements delineate the principal hydrothermal alteration patterns across the study area.

Remote sensing has been extensively applied in mineral exploration due to its capability to detect hydrothermal alteration zones, whose associated minerals exhibit diagnostic spectral characteristics in the VNIR and SWIR regions. OH-bearing minerals display prominent absorption features near ~ 2.2 μm, while iron oxides show characteristic absorption in the blue wavelengths and enhanced reflectance in the red region^40,103,104^. However, the presence of these minerals alone does not necessarily indicate economic mineralization, as similar spectral signatures may result from weathering. Consequently, hydrothermal alteration zones in this study were delineated based on the spatial coincidence of VNIR-derived iron oxide anomalies and SWIR-derived OH-bearing mineral anomalies, supported by magnetic data analysis, geological context, existing maps, and known mining occurrences. This integrated interpretation enhances the reliability of the alteration mapping and its relevance to mineral exploration.

The spatial comparison between the PRISMA-derived maps in Fig. 10a and b and the known mining sites in the study area demonstrates a clear spatial coincidence. Documented mining locations, illegal mining activities, coincide with PRISMA-detected alteration zones, particularly those characterized by diagnostic minerals such as OH-bearing and iron-bearing alteration minerals. These minerals are commonly associated with hydrothermal processes responsible for gold mineralization in the region. The observed correspondence not only validates the reliability of PRISMA data for mineral detection but also emphasizes its potential for identifying new prospective zones beyond currently exploited sites.

### Aeromagnetic data analysis

**Fig. 11:**
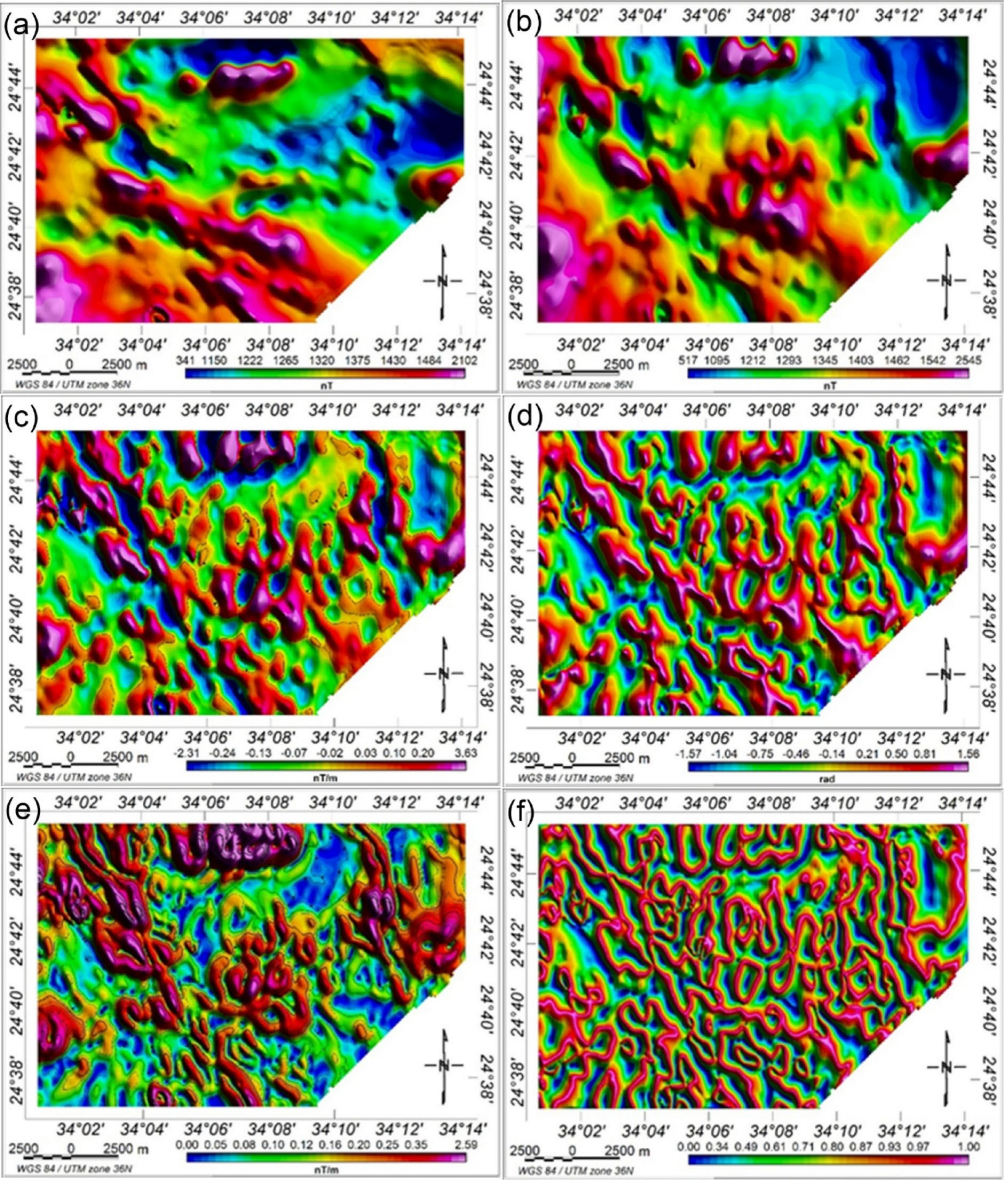
Shaded color of (**a**) TMI map, (**b**) RTP magnetic map, (**c**) the FVD map, (**d**) TA map, (**e**) HGM map, and (**f**) TM map.

Aeromagnetic data were used in the present analysis to investigate the structural patterns of the region under consideration. The TMI map (Fig. 11a), acquired from Aero-Service (1984), was processed using a RTP filter to produce the RTP magnetic map (Fig. 11b). To enhance the signal-to-noise ratio and reduce the influence of the noise, the aeromagnetic data, encompassing all the research area’s magnetic anomalies, were upwardly continued to 50 m before applying the chosen filters. In potential field geophysics, upward continuation is a method of filtering used to improve magnetic or gravity data by calculating the field values at a higher altitude than the original survey level. This simplify the identification and interpretation of geological structures that might be obscured by anthropogenic features introducing noise into the data^105–107^.Visualization of general hydrothermal alterations 

The research area’s RTP magnetic map displays both high and low amplitude anomalies with a range of 517 to 2445 nT. Figure 11b shows that strong-amplitude magnetic anomalies are almost concentrated to the southwestern, northwestern, eastern, and central parts of the region under investigation, whereas low-amplitude magnetic anomalies are situated in the northeastern portion. The study area’s central region is dominated by a broad belt of long-wavelength positive anomalies extending toward the northwest-southeast. In the southern part, significant anomalies trend north–south, while the northeastern region is dominated by regional low-amplitude anomalies oriented north–south. Additionally, the northern area exhibits high-frequency, nearly E–W trending positive magnetic anomaly. The overall geometry of magnetic anomalies indicates prevailing structural trends in the NW–SE, E–W, NE–SW, and N–S directions, reflecting regional structural framework as depicted in the geological map (Fig. 1). These observations may indicate that residual magnetization has a minimal or negligible influence on the magnetic properties of the local sources.

The FVD map of the RTP magnetic data (Fig. 11c) consists of anomalies with varying frequencies and can be helpful in highlighting fault trends and delineating the significant bodies’ borders. Low-frequency anomalies are linked to deep sources, while high-frequency anomalies indicate shallow basement structures. Major fault lines, represented by zero contours in the FVD, cut through various subsurface units. Three primary structural trends form the main the structural pattern in the study area. These trends are NW–SE, NE–SW and N–S.

The TA of the RTP magnetic data is shown in Fig. 11c. Structural resolution is highest at locations where TA values approach zero near the anomaly edges (Fig. 11d). The amplitudes of the angles range from − 1.57 to + 1.56 rad, corresponding to angles between − 90° and + 90°. Weak magnetic anomalies that can be hidden by greater ones are amplified by the TDR filter^19^. This map displays several buried features not visible in the geological map. As a result, it is possible to assume that three main sets of faults striking NW–SE, N–S, and E–W. have displaced basement rocks.

The HGM map (Fig. 11e), obtained by applying Eq. 2 to the RTP magnetic data, shows that the HGM maxima correspond to geological boundaries and faults, particularly at shallow depths. The HGM method identifies numerous linear features and shallow geologic structures, with the NW–SE, NE–SW, E–W, and N–S directions being most prominent. While the HGM method effectively maps shallow structures, it is less sensitive to deep-seated features.

Results of TM technique applied to the RTP magnetic data are shown in Fig. 11f. The TM approach allowed a sharper detection of geological characteristics in respect to the other methods and was very successful in balancing magnetic anomalies characterized by different amplitudes. However, its interpretation in the Wadi Shait area is limited due to the interconnected nature of detected edges, making structural analysis challenging. The TM technique, relying on vertical gradient zero-crossings, also produces some spurious contours around source bodies^108^.

**Fig. 12:**
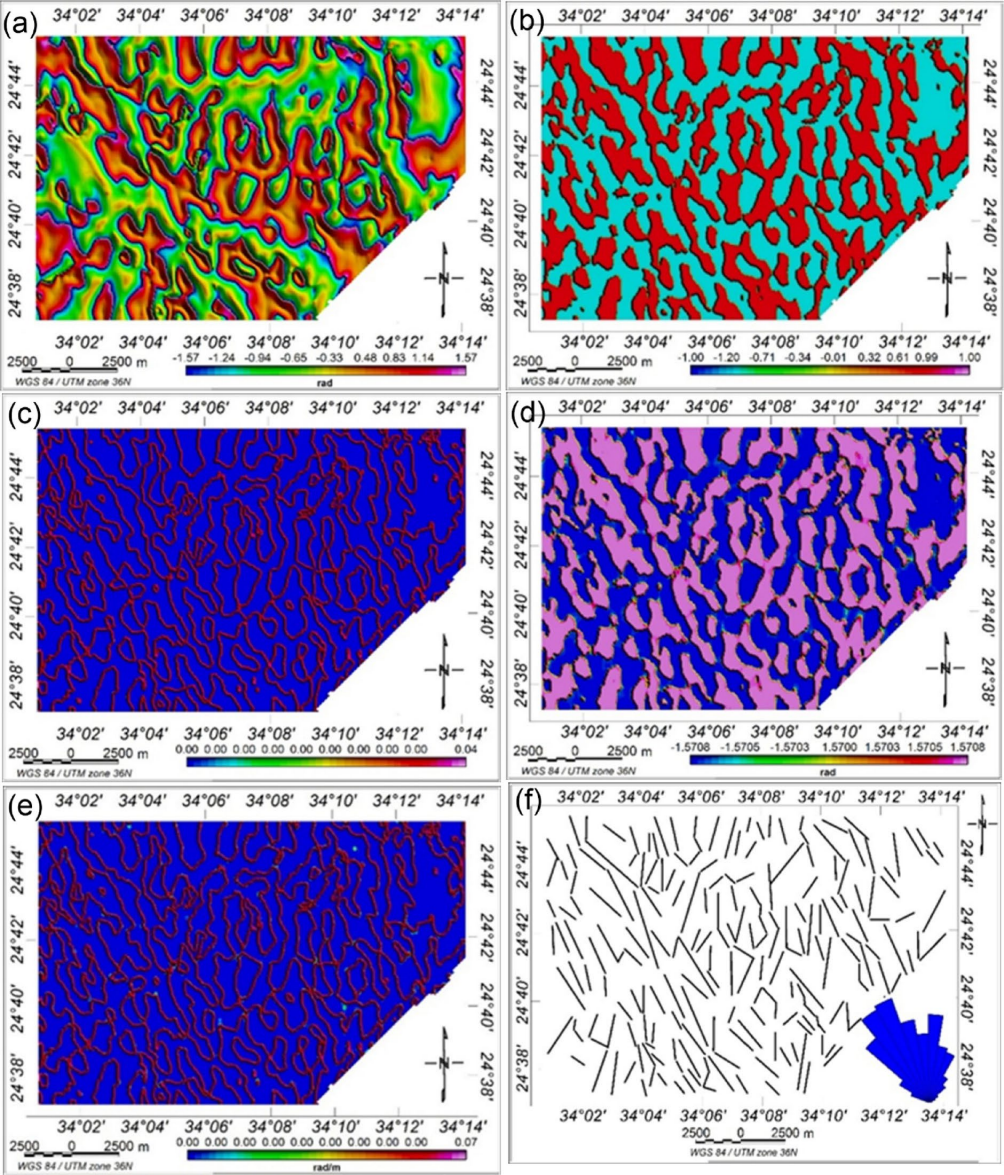
(**a**) TDX map, (**b**) impTDX map, (**c**) THDR_impTDX map, (**d**) STDR map, (**e**) THDR_STDR map, and (**f**) Interpreted structural map of the study area deduced based on the results of these filters. The rose diagram of the lineaments is shown in (**f**), where the NW–SE is the main tectonic trend in the area.

Figure 12 show the application of advanced edge detection techniques to the RTP magnetic data, including TDX, impTDX, THDR_impTDX, STDR, and THDR_STDR filters. Filters such as impTDX and THDR_impTDX effectively image previously undetectable structures. These structural boundaries are likely related to basement intrusions, faults, and lithologic contacts. Four dominant structural trends, NNW–SSE, NW–SE, NE–SW, and N–S, shape the regional framework. The STDR filter (with M set to the average magnetic field strength, 40,806 nT) (Fig. 12d) and its horizontal derivative (Fig. 11e) effectively normalize strong and weak anomalies of varying depths, allowing enhanced edges of the subsurface magnetic sources. These maps improve the clarity of magnetic anomaly borders, facilitating a more accurate interpretation of the potential field data.

**Fig. 13:**
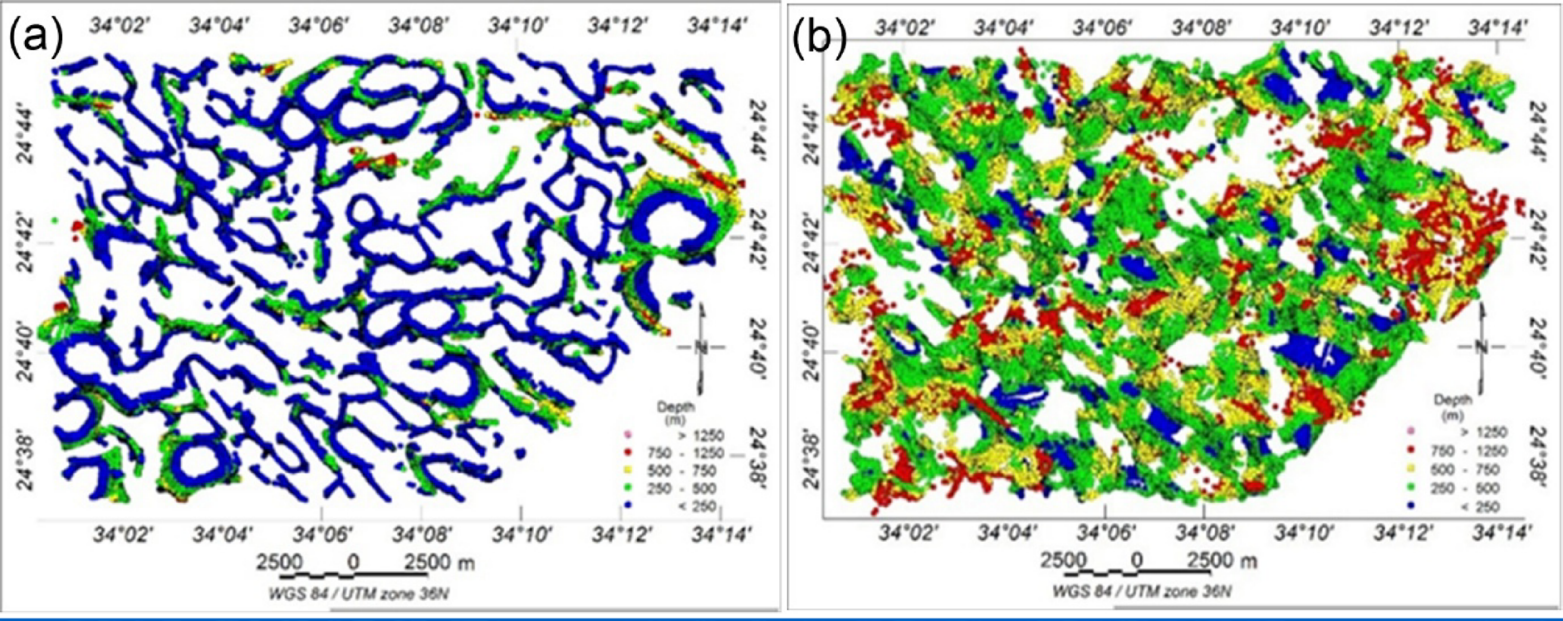
(**a**) The depths to the magnetic sources obtained by the ED method with a window size (10 × 10), obtained by structural index zero and (**b**) structural index one.

**Fig. 14:**
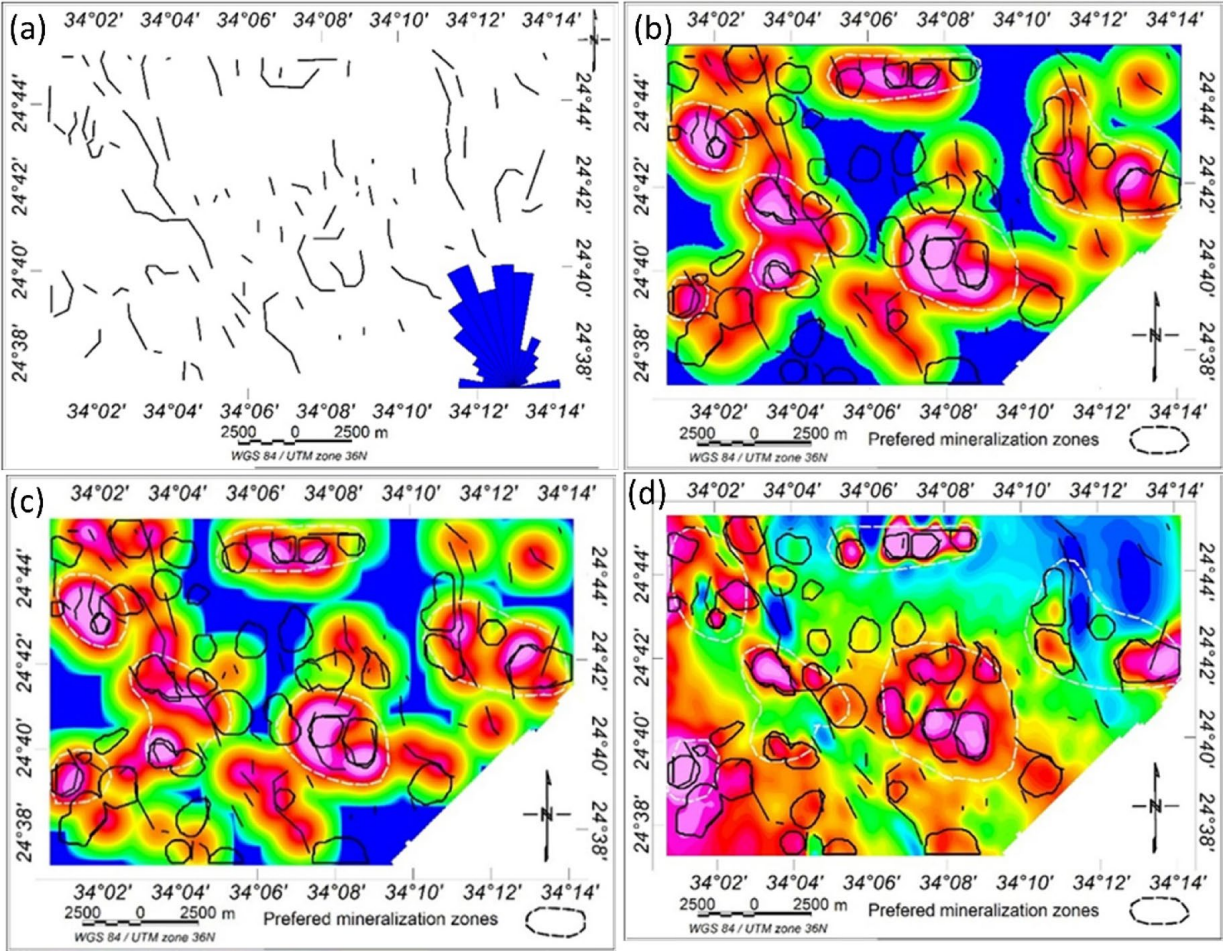
(**a**) The structural complexity map of the study area from the CET grid analysis inserting rose diagram, the circular boundary porphyry tracing (blue color) and skeleton of vector lineaments (black lines) are superimposed on (**b**) the Contact occurrence density (COD) map, (**c**) Orientation entropy heat map (OE) map, and (**d**) RTP magnetic map.

The ED results (Fig. 13a, b) were computed using structural indices of 0 and 1 to delineate the positions and depths of subsurface structures. Color dots represent source edges across five depth intervals, showing good agreement with known faults and contacts. ED clusters in Fig. 14a (structural index 0) mark shallow magnetic contacts (< 250 m), with some deeper sources (> 750 m). In Fig. 13b, clusters represent faulted rock edges, dikes, and sills. The NW–SE, NE–SW, N–S, and E–W trends are clearly delineated. The deepest structures are generally around 250 m.

The CET grid analysis technique, which comprises multiple processes including texture analysis, lineation detection, and lineation vectorization was applied to the RTP magnetic grid to generate the structural complexity map of the study area, as shown in Fig. 14a. This analysis allowed for the identification of the most prominent structural patterns, both at the surface and at depth. Notably, the dominant structural trends inferred from the CET analysis are NW–SE, NE–SW, and N–S.

To further delineate zones of mineralization potential, two key derivative products were produced: the contact occurrence density (COD) map and the orientation entropy (OE) heat map. These maps help highlight areas with dense structural intersections and complex geological settings, which are often favorable for mineral deposition. Porphyry detection was performed using the circular feature transform (CFT) to detect circular structures, followed by center peak identification and amplitude contrast mapping.

The boundaries of circular porphyry-like features (in blue) and the skeleton of vector lineaments (in black) were superimposed onto the COD map, the OE map, and the RTP magnetic map (Figs. 14b–d). These maps clearly exhibit the dominant NW–SE and NE–SW trends in the Wadi Shait mining area. The integration of these layers reveals that the identified contacts correspond to zones of structural orientation changes and the intersections of multiple lineaments. In particular, the red zones on the COD map (Fig. 14b) indicate potential deposit-hosting locations. The OE map (Fig. 14c) highlights regions with high structural complexity, characterized by scattered or disordered structural orientations. Additionally, the superposition of porphyry outlines and structural skeletons on the RTP magnetic map (Fig. 14d) shows that preferred mineralization zones often align with areas of high magnetic anomaly.

Structural complexity analysis, as demonstrated by Holden, et al. ^96^, is a valuable tool for locating favourable ore deposits. Here, the CET grid analysis proved effective in delineating geological boundaries and in identifying promising zones for mineral exploration. However, it is important to note that the CET technique did not detect all known mineralized areas; some documented occurrences lie within low-entropy zones (the blue areas in Figs. 14b-d), which were not classified as favourable by the CET analysis.

**Fig. 15:**
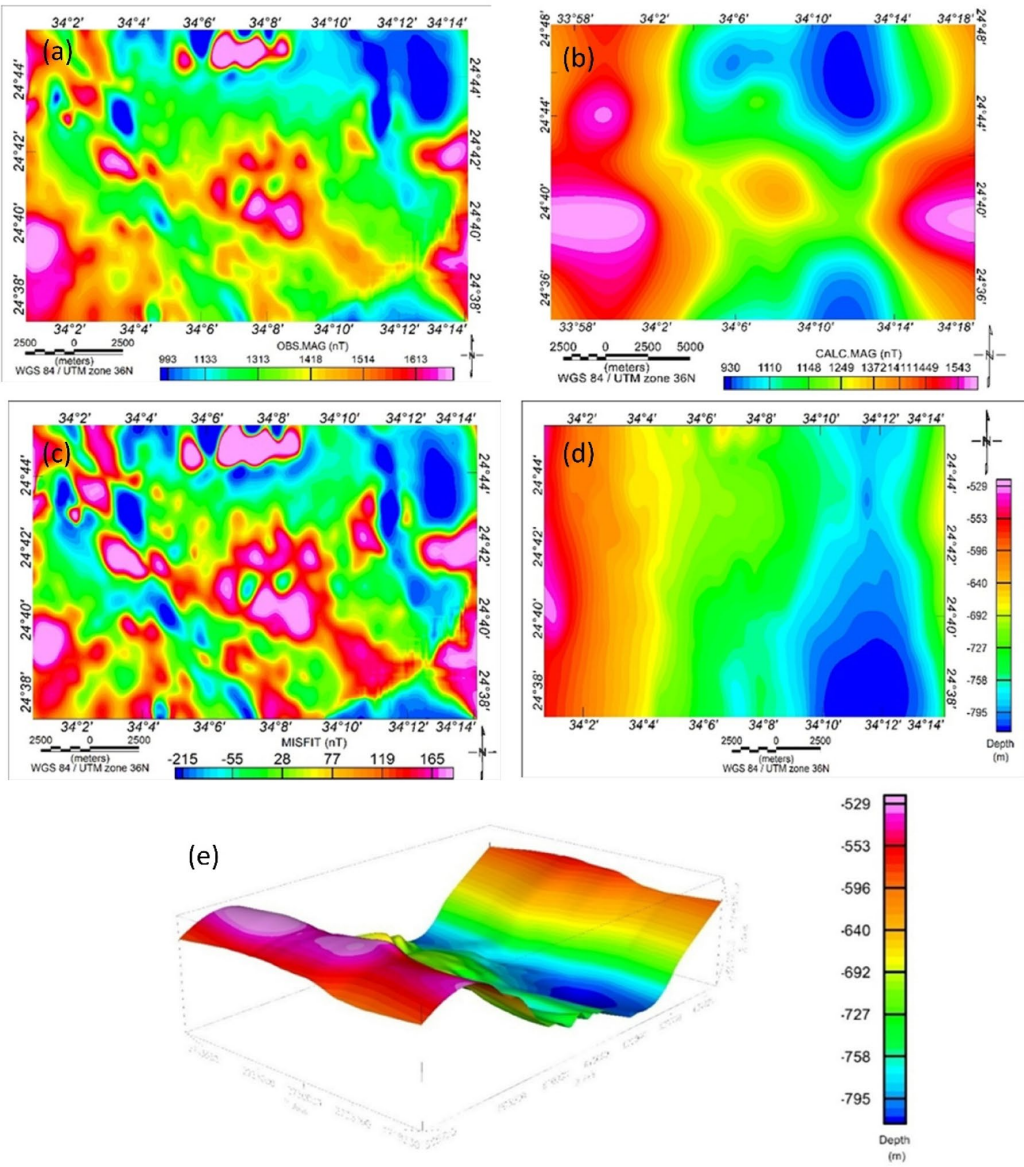
3-D view map. Results of 3D inversion of magnetic data, which include (**a**) observed RTP magnetic map, (**b**) calculated magnetic map, (**c**) data misfit, (**d**) inverted depths to basement rocks, and (**e**) 3D perspective showing the topography and the depths to basement rocks. Aeromagnetic data are processed, and maps are produced by Geosof Oasis Montaj sofware v. 7.0. https://www.seequent.com/products-solutions/geosof-oasis-montaj.

Following the confirmation of geological consistency, a 3D subsurface model was constructed to visualize the basement geometry and support further interpretation. Within this framework, forward magnetic modeling of shallow magnetic structures was performed using one of the most established techniques in magnetic interpretation, 3D forward modeling. The GMSYS-3D was used to perform the magnetic modeling on the observed TMI data (Fig. 15). This helped estimate the depth to the top of the basement surface^6,109–111^.

The modeling grid was defined with 404 cells in the X-direction and 269 cells in the Y-direction, with a grid cell size of 100 m. All grids were projected consistently and shared the same distance units, dimensions, and resolution. The 3D magnetic model consisted of a set of stacked grid surfaces, with subsurface layers defined in terms of magnetic susceptibility and magnetization properties. The calculations were performed using the Parker method^101^ in the wavenumber domain.

A two-layer subsurface model was assumed: the upper layer comprising non-magnetic sedimentary rocks, and the lower layer representing magnetic basement rocks. The magnetic susceptibility of the basement rocks was estimated at approximately 0.125663 SI (0.01 cgs), while the sedimentary rocks were considered to have negligible (zero) magnetic susceptibility. The model also incorporated the magnetic field parameters relevant to the study area, including a total field strength of 40,806 nT, an inclination of 34.45°, and a declination of 1.93°.

The model performed several iterations by adjusting the initial input magnetic susceptibility, gradually reducing the misfit between the calculated and observed data. This iterative process continued until the model achieved an acceptable error margin. The final results showed a good correlation between the observed and calculated magnetic data (Fig. 15a and b), with a satisfactory misfit (Fig. 15c). The resulting basement relief and 3D view maps (Fig. 15d and e) indicate that the basement surface depth varies from less than 529 m in the western part of the study area to around 795 m in the southern and eastern parts. These results are consistent with those obtained from the ED and SPI methods, thereby enhancing the reliability of the basement structure interpretation.

### Field structural observations and ground validation

**Fig. 16:**
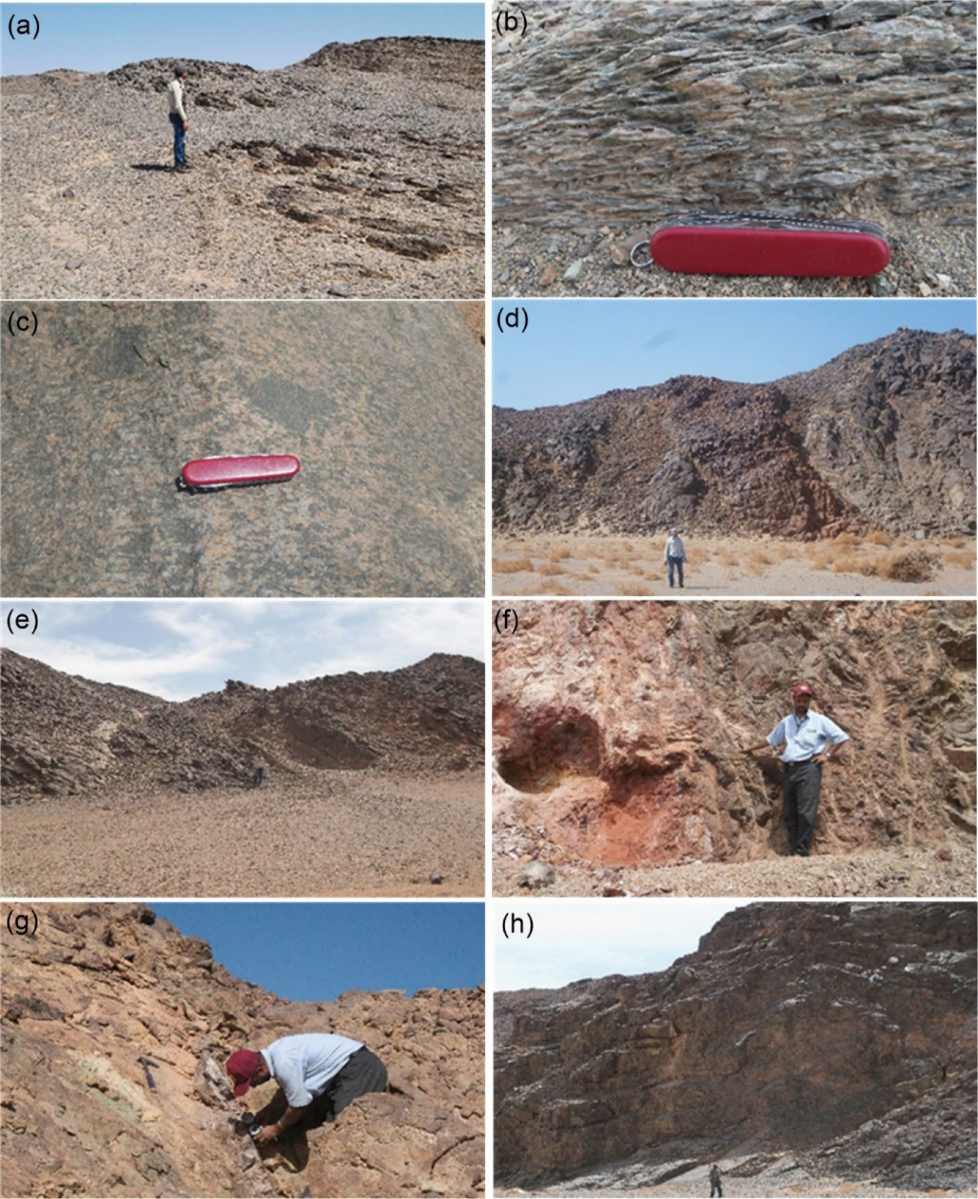
(**a**) General view looking NE, showing intensive erosion of NE-dipping metasedimentary slices forming part of the hinge zone of a major antiformal structure in the GOM. (**b**) Field photograph of S-C shear fabrics showing the NW-directed tectonic transport of the GOM over the SGC. Photograph taken at the northeast part of the second occurrence of the GOM. (**c**) Photograph at the southern margin of the SGC showing incorporation of schistose non-oriented mafic xenoliths. (**d**) General view showing intrusions of monzogranite (reddish brown) into tonalite rocks (grey). (**e**) General view of the eastern part of the SGC showing imbrication of the complex into NE-dipping slices with a remarkable crude foliation. (**f** and **g**) General views of a NW-SE-trending sinistral shear zone, featuring an old shaft, initial mining operations, and an open pit with auriferous zones. These zones include deformed quartz veins containing malachite and iron-bearing materials. (**h**) Field photograph of Dokhan volcanics showing basalt intrusion at the lowermost section into andesite rocks. All photographs are original and were taken by the authors. The person shown in panels (**d**), (**f**), and (**g**) is one of the authors.

Detailed field investigations document intense deformation within the GOM. In the southern exposure, rocks are intersected by a prominent NW-SE-trending dextral strike-slip fault system, along which foliation is well developed and stretching lineations plunge 10–15° toward the NW. This exposure forms the eastern limb of a major antiformal structure characterized by NW-trending fold axes with moderate plunge. In the northern exposure, foliation dips steeply toward the north and northwest, and step-like normal faults dipping 70–80° northward define tectonic contacts with adjacent units. Thrusting of the GOM toward the NW over the SGC is supported by S-C shear fabrics, asymmetric folds, and stretching lineations (Fig. 16a, b).

The intrusive relationship between the SGC and the GOM is confirmed by granite off-shoots penetrating the mélange and by a narrow interaction zone composed of mixed granite and mélange material along the southern bank of Wadi Shait. Numerous enclaves of metabasalts and metagabbros derived from the mélange occur within the granite mass, displaying irregular boundaries and variable degrees of assimilation (Fig. 16c). Additional evidence includes irregular granite xenoliths within marginal intrusive zones, further supporting magmatic emplacement.

Within the SGC, the outer zone exhibits pervasive NW-trending gneissic foliation and stretching lineation. These fabrics are spatially associated with NW-trending dextral strike-slip shear zones that progressively transform tonalitic and granitic rocks into protomylonites, mylonites, and locally ultramylonites (Fig. 16d, e). Deformation intensity in the outer zone is markedly greater than in the core.

In the Hamash granodiorite, two principal quartz vein types are recognized. The first consists of large smoky quartz veins enriched in iron oxides and malachite, whereas the second comprises smaller quartz veins and veinlets. These veins are structurally controlled, trending N-S and dipping westward, and are associated with hydrothermal alteration (Fig. 16f, g).

Within the Dokhan volcanics, xenoliths derived from the SGC and Hamash granodiorite confirm interaction between these units. Additionally, angular gneissose granite inclusions occur within Younger Granite bodies near their contacts with the SGC, supporting their intrusive nature (Fig. 16h).

## Structural framework

To accurately characterize and interpret the geological framework of the Wadi Shait region, we integrated field geology, remote sensing, and aeromagnetic geophysical data. Magnetic lineament analysis based on the RTP magnetic anomaly map included the production of multiple derivative and enhancement grids such as impTDX, STDR, FVD, TA, HGM, and TM as well as advanced techniques including 3D ED, SPI, and CET grid analysis. These tools were employed to delineate key fault structures, shear zones, and fracture systems shaping the region’s tectonic architecture. Magnetic lineaments were interpreted to represent primary fault zones, fractures, and shear corridors, enhancing our understanding of the tectonic forces that governed the deformation history of the area.

According to the geological map (Fig. 1) and prior studies^3,41,42,45,47,112^, the study area lies within the southwestern segment of the Idfu–Marsa Alam shear zone, specifically along the Wadi Bezah shear zone^113^ a major ENE to E–W trending right-lateral strike-slip corridor. The region exhibits a complex array of tectonic structures, including NW–SE dextral strike-slip faults, NE–SW normal faults, and subordinate N–S-trending faults.

**Fig. 17:**
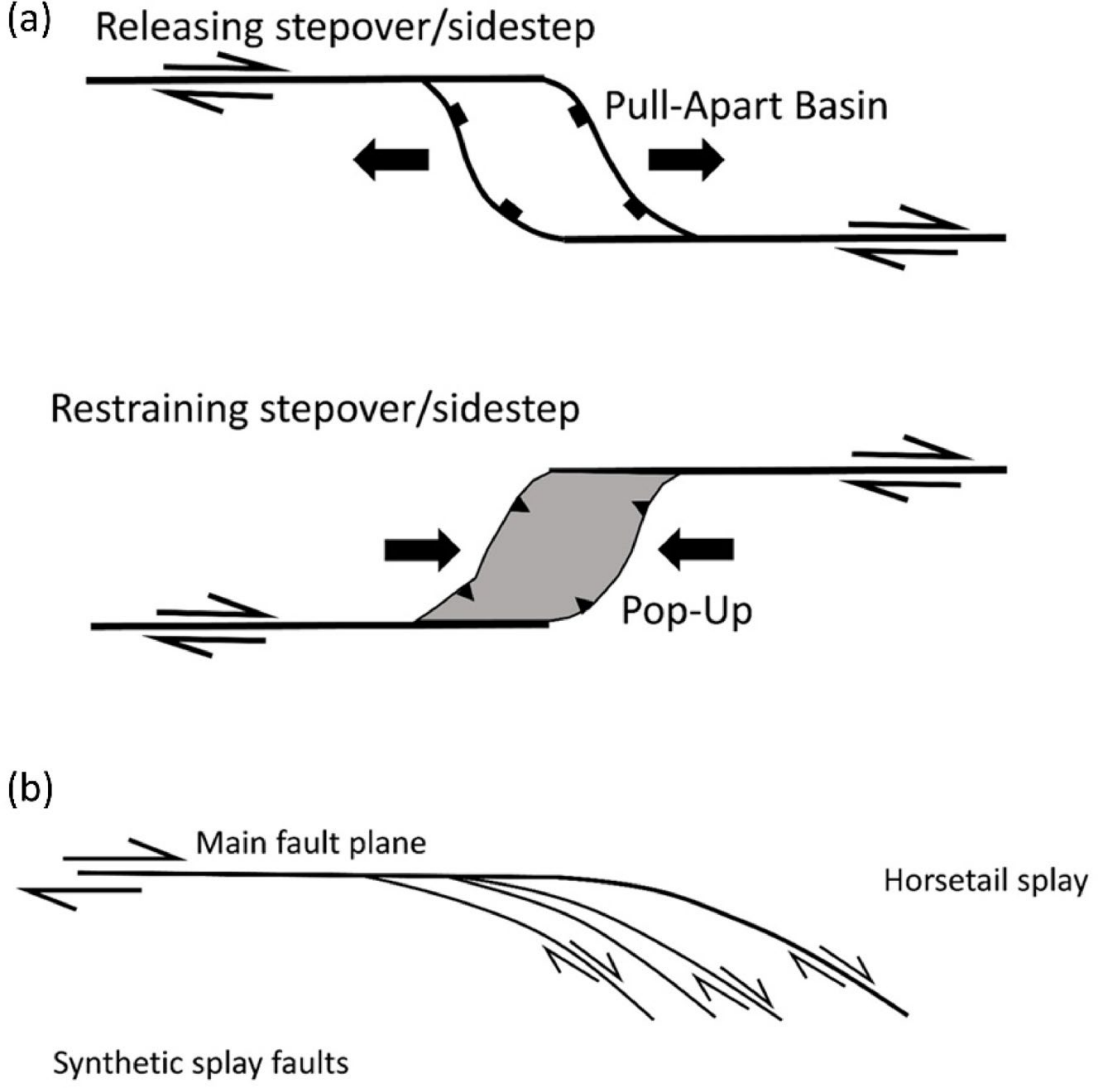
(**a**) General characteristics of strike-slip fault systems in plain view after Dooley and McClay^121^. (**b**) A sketch showing a horsetail splay structure after Cunningham and Mann^115^.

Notably, a releasing bend developed along WNW-trending dextral strike-slip faults (Fig. 17a) generated localized extensional zones that facilitated granite emplacement and associated hydrothermal gold mineralization. These dilational sites served as conduits for granitic melts ascending under reduced lithostatic pressure and subsequently intruding into the upper crust. Simultaneously, the extensional regime increased permeability, focusing hydrothermal fluid flow from crystallizing magmas and leading to gold precipitation within fractures, veins, and brecciated fault zones^114,115^. Continued activity of these structures sustained prolonged fluid flux and metal deposition, with mineralization localized in late-stage brittle structures overprinting earlier ductile fabrics^116,117^.

In places, the dextral strike-slip fault system exhibits a geometry consistent with a horsetail splay (see highlighted area in Fig. 1b and detailed view in Fig. 5b; schematic representation in Fig. 17b), characterized by fan-like branching of subsidiary faults near fault terminations. Such structures accommodate strain during fault propagation or curvature and generate zones of distributed brittle deformation^118^. These inferred horsetail splays underscore the segmented and evolving nature of strike-slip systems and their mechanical interaction with surrounding host rocks^119,120^.

The older units, including the SGC and the GOM, have undergone brittle to ductile deformation and are crosscut by NW-trending shear zones (Fig. 1a), ranging from several meters to tens of meters wide. These units form part of the broader fault-and-thrust belt of the central and southern Eastern Desert^41^. The SGC itself forms a prominent antiformal structure, akin to domes in the Meatiq and El-Sibai regions^55^. Its exhumation and extension are closely tied to Najd-related oblique transpressional faulting, characterized by NW-trending stretching lineations and NE- to SW-dipping foliations.

**Fig. 18:**
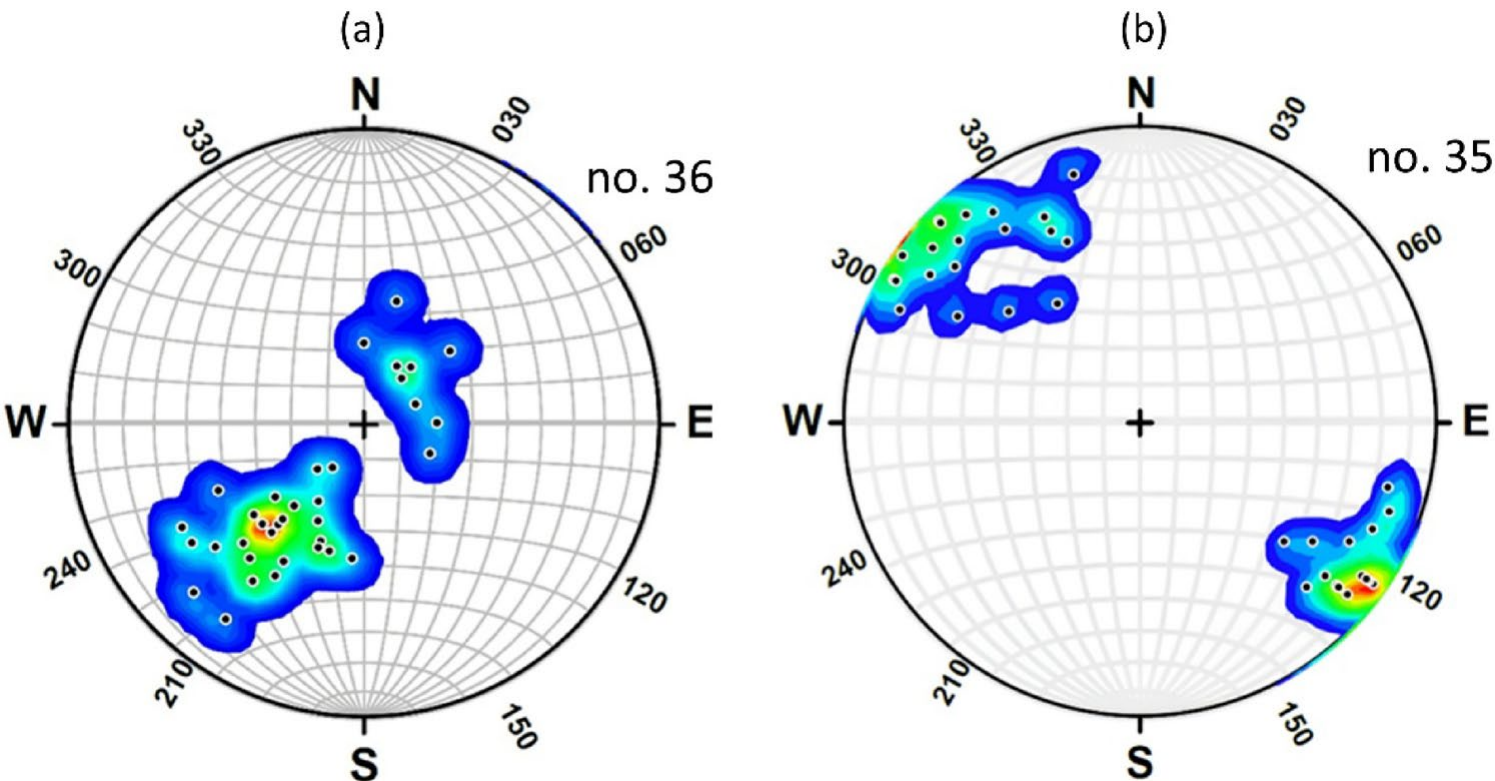
(**a**) Lower hemisphere equal-area stereographic projections of 36 planes and their poles contouring of the major antiformal structure. (**b**) Lower hemisphere equal area projection of 35 mylonite lineation points and their contouring.

Foliation in the eastern limb strikes NW-SE and dips 10°-75° NE. Two principal clusters of stretching lineations are identified. The dominant cluster trends approximately 315°-330° and plunges 10°-25° toward the NW, whereas a secondary cluster trends 125°-140° and plunges 10°-30° toward the SE. The western limb, although less exposed due to down-faulting beneath the Nubia Sandstone, exhibits foliation striking NW-SE and dipping 10°-30° SW. Lineations in this limb show comparable orientations, trending NW–SE and plunging 5°-45° toward both NW and SE directions (Fig. 18a, b).

Regional E-W constriction associated with Najd-related transpressional deformation likely resulted in stress partitioning and localized extensional collapse along the SGC margins, leading to the development of steeply dipping normal faults (Fig. 1a). These NE-dipping faults in the western sector dip away from the dislocated basement blocks (Fig. 1c) and likely predate the unconformable deposition of the Nubia Sandstone.

Structurally, the region is governed by four main tectonic trends: NW-SE, NE-SW, N-S, and E-W. The NW-SE trend, attributed to the Gulf of Suez-Red Sea rifting and Najd fault systems represents the dominant structural orientation^122^. The NE-SW trend, associated with the Gulf of Aqaba-Dead Sea system, reflects rejuvenated pre-Cambrian shearing due to Arabia’s northward motion^123^. The N-S trend corresponds to the East African–Nubian structural grain^124,125^, related to the Mozambique Belt and Pan-African orogeny^126^. The less prominent E-W trend, known as the Tethyan trend, represents one of the earliest tectonic influences on the Egyptian Precambrian basement^1^.

Our geological interpretations are founded upon a robust integration of multi-disciplinary datasets. Remote sensing data provided the initial framework for lithological and structural mapping, identifying key trends that were subsequently validated and refined through other datasets. For instance, the east-west (northern part of the study area) and northwest-southeast (southern part of the study area) trending lithologies observed in remote sensing were powerfully corroborated by distinct patterns in the aeromagnetic data, particularly evident in the TMI and RTP magnetic maps where high magnetic values align precisely with these delineated trends.

These remotely and geophysical defined units were then subjected to rigorous field validation, confirming their distinct lithological characteristics, which were further substantiated by variations in geochemical signatures. This multi-scale approach ensured that initial interpretations from one dataset were systematically cross-referenced and strengthened by evidence from others.

Furthermore, the structural framework of the study area was established through a convergent approach. Visual analysis of remote sensing imagery revealed prominent NE, NW, and EW structural trends, particularly in the orientation of major wadis. These structurally controlled features were directly confirmed and enhanced by aeromagnetic lineament analysis, derived from techniques such as improved horizontal tilt angle (impTDX) and STDR Filter and the total horizontal derivative of these filters, First Vertical Derivative (FVD), Horizontal Gradient Magnitude (HGM), and Tilt Derivative (TDR).

The consistent alignment between these diverse datasets from regional-scale remote sensing and aeromagnetic to site-specific fieldwork underscores a cohesive interpretative methodology. This integrated approach allowed for the development of a comprehensive geological model where each data type contributed uniquely to, and simultaneously validated, the overall interpretation of the area’s geology and structural evolution.

It should be emphasized that the final output of this work represents a productivity map deduced from an updated geological map of the study area (Fig. 21), which was field validated through two main field campaigns and aeromagnetic data. During the validation process, lithological units and key lithological contacts were systematically checked in the field. The locations of the field stations were superimposed on the geological map together with the major structural features, as illustrated in Fig. 20. Furthermore, based on the integration of remote sensing, and structural interpretation, the main hydrothermal alteration zones were validated using the spatial distribution of known mining sites within the study area. This confirms the reliability of the detected alteration zones, which in many cases spatially coincide with major structural features, highlighting the structural control on gold mineralization. In addition, field-visited alteration zones (Fig. 20) are presented to further support and validate the obtained results, as shown in Fig. 21.

**Fig. 19:**
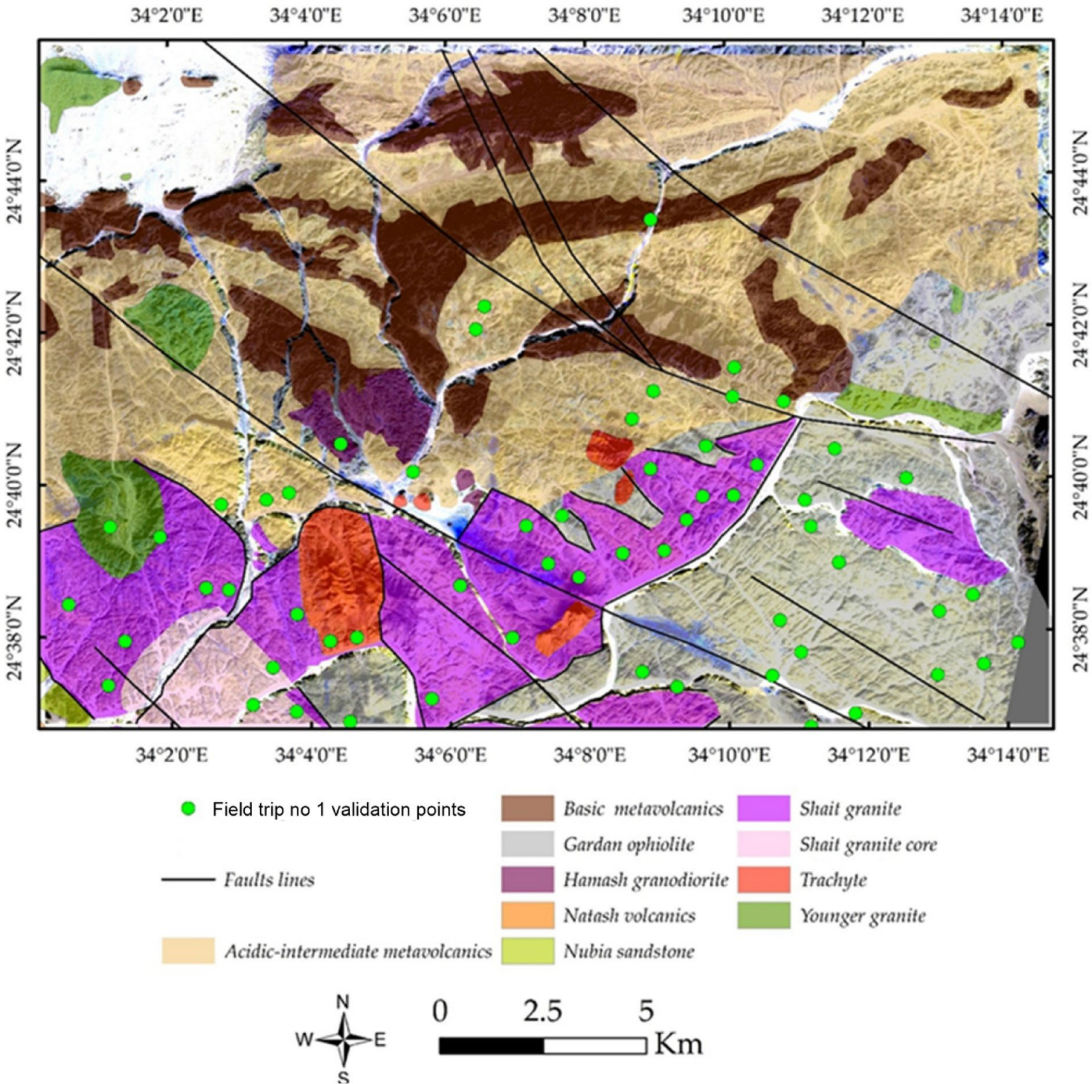
Updated geological map of the study area illustrating lithological units and major structural features, field-validated through the spatial distribution of field stations overlaid on the map. The figure was created using ENVI v. 5.6.2 software (https://www.l3harrisgeospatial.com/Software-Technology/ENVI).

**Fig. 20:**
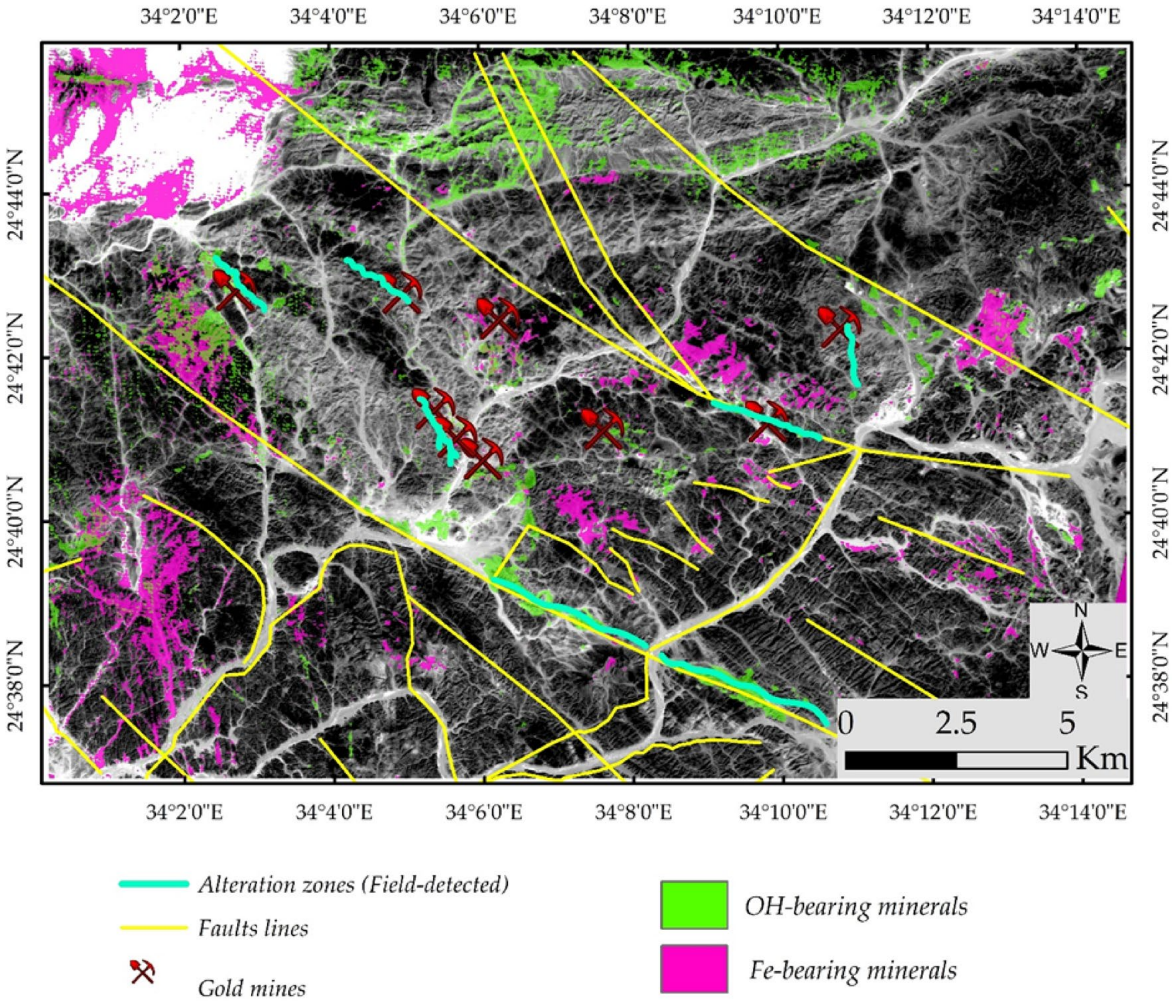
Spatial distribution of hydrothermal alteration zones derived from remote sensing data and structural elements, with validation based on known mining sites and field-visited alteration localities, confirming the structural control on gold mineralization.

**Fig. 21:**
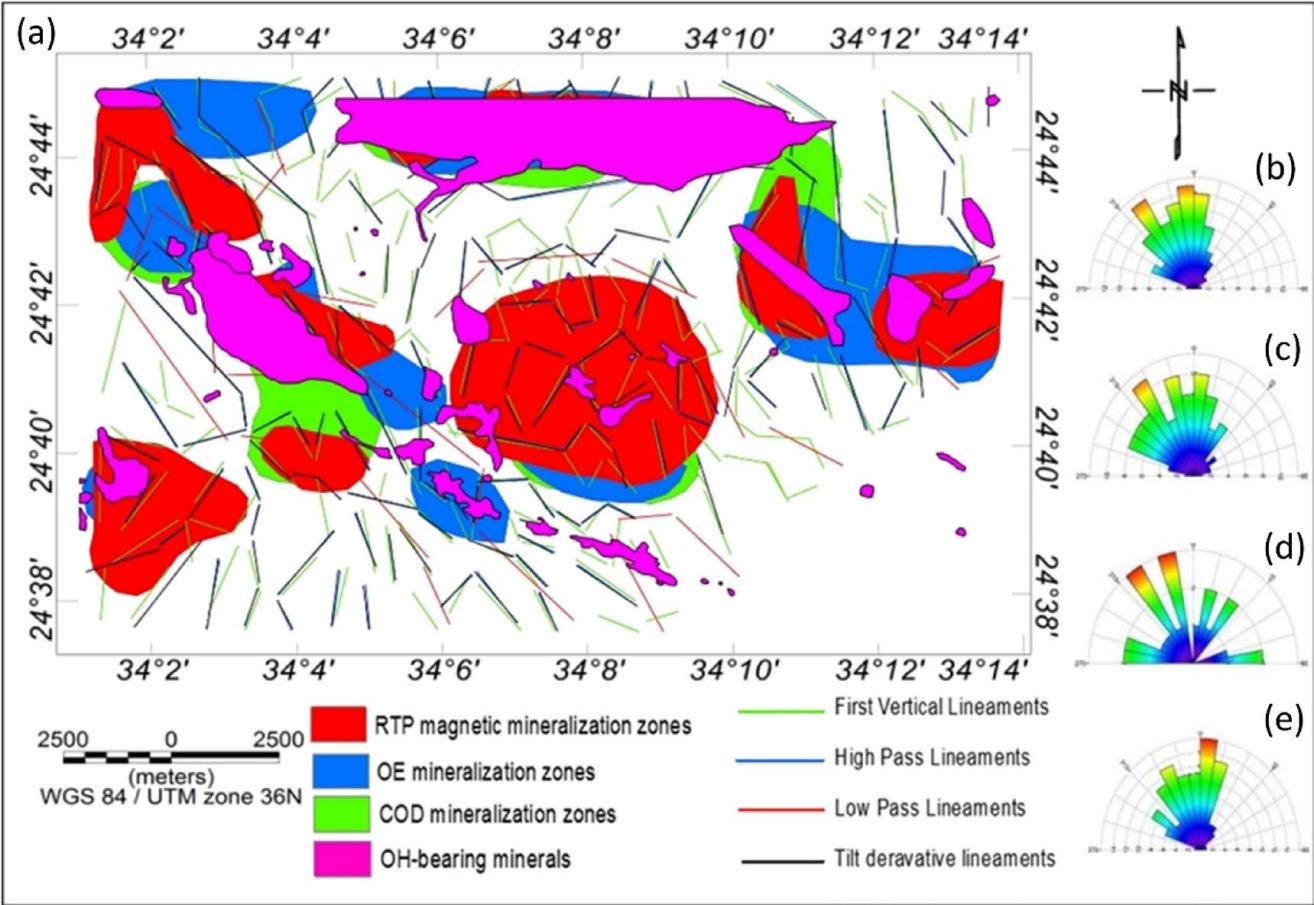
(**a**) Lineaments structural map of the study area detected from FVD, HP, LP, and TDR filters. Rose diagrams obtained from (**b**) HP, (**c**) LP, (**d**) FVD, and (**e**) TDR maps. Created using ArcGIS Desktop v 10.3. https://www.esri.com/en-us/arcgis/products/arcgis-desktop/overview. Rose Diagrams created using RockWare.RockWorks.15.Complete.v2011.7.6. https://www.rockware.com/product/rockworks/.

## Conclusion

This study integrated field observations, remote sensing, and aeromagnetic data to characterize the GOM and SGC in the Wadi Shait area. The research area’s mineralization is substantially structurally regulated, as shown by integrated aeromagnetic and PRISMA remote sensing investigations. Major fault and shear systems that serve as the main routes for hydrothermal fluid migration are defined by four prominent structural orientations seen in aeromagnetic data. The results show NW-directed thrusting of the GOM over the SGC, which exhibits extensive mylonitization related to Pan-African deformation.

Aeromagnetic analysis identified four dominant structural orientations (NW–SE, NE–SW, N–S, E–W) with source depths between 124 and 782 m, while 3D magnetic modeling revealed basement depth variations from 529 to 795 m. Remote sensing with Sentinel-2 imagery proved effective in distinguishing lithological units, mapping structures, and detecting potential hydrothermal alteration zones. Mineral prospectively is further limited by differences in basement depth; zones of increased fracture and fluid concentrating are represented by shallow basement domains and basement highs. High COD and orientation entropy values identify structurally complicated corridors that align with alteration zones rich in iron oxide and hydroxyl, indicating their function as advantageous mineralization sites. Discrete, high-priority exploration sites are defined by the spatial convergence of structural complexity, shallow basement architecture, and hydrothermal alteration. These findings create a cohesive exploration model that supports target creation and mineral resource assessment by combining aeromagnetic structure, crustal architecture, and remote sensing alteration data.

These findings highlight the structural framework and alteration patterns that control the distribution of gold and radioactive minerals, thus enhancing mineral exploration potential in the region. Future work should incorporate geochronology, high-resolution geophysical surveys, and mineralogical isotopic studies of alteration zones. The integrated methodology presented here offers a robust framework for studying complex geological terrains and guiding exploration strategies.

## Supplementary Information

Below is the link to the electronic supplementary material.

Supplementary Material 1

## Data Availability

The datasets used and/or analysed during the current study are available from the corresponding author upon request.
