# Supplementary material for: Multidisciplinary approaches to lithological discrimination and structural mapping for mineral resource assessment
Source: Sci Rep. 2026 Mar 13;16:9079. doi: 10.1038/s41598-026-43824-x (PMC12993058; doi:10.1038/s41598-026-43824-x)
Supplement: Supplementary file 1 — Supplementary Material 1 [file 41598_2026_43824_MOESM1_ESM.docx]

**Supplementary Figure S1. Spatial distribution of field sampling sites in the Wadi Shait area.**

Each point represents one sampling locality from which one or more rock specimens were collected (total: 139 sites, ≈ 400 samples).


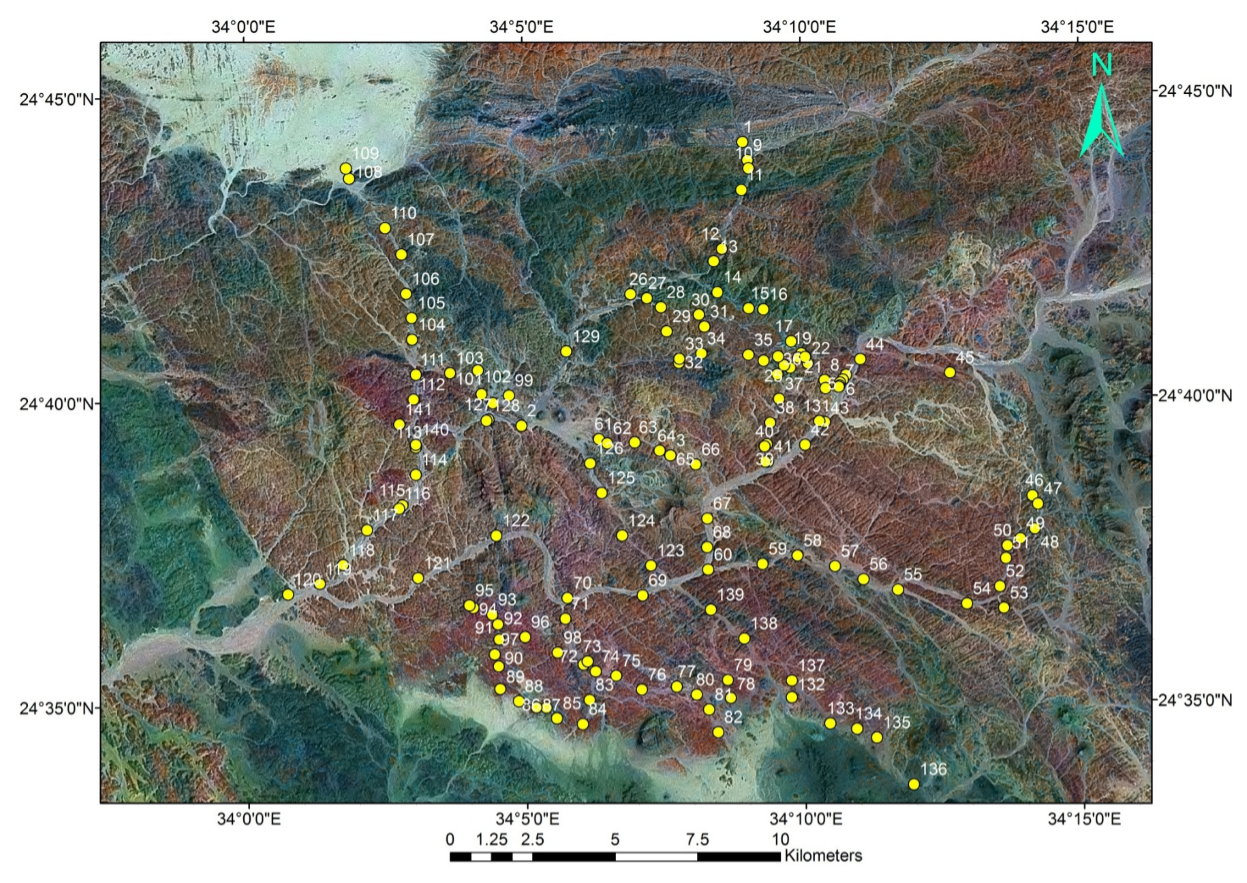


| **Supplementary Figure S1.** Geographic distribution of 139 field sampling sites in the Wadi Shait area projected on a Landsat 7 (ETM+) image (USGS EarthExplorer, public domain). Each point corresponds to a sampling locality from which multiple rock specimens were collected. The figure was created by ArcGIS Desktop v 10.7.1. software; (<https://www.esri.com/en-us/arcgis/products/arcgis-desktop/overview>). |
| --- |
